# Impact of SARS-CoV-2 RBD Mutations on the Production of a Recombinant RBD Fusion Protein in Mammalian Cells

**DOI:** 10.3390/biom12091170

**Published:** 2022-08-24

**Authors:** Guillaume Gerez, Jerome Martinez, Christophe Steinbrugger, Sandra Bouanich, Johanna Dimino, Corine Piegay, Maxime Combe, Franck Berthier, Soizic Daniel

**Affiliations:** BioMérieux, R&D ImmunoAssays, 69280 Marcy l’Étoile, France

**Keywords:** SARS-CoV-2, RBD, E484K mutation, disulphide bond, chaperone, protein disulphide isomerase, endoplasmic reticulum, misfolding, protein yield, mammalian cells

## Abstract

SARS-CoV-2 receptor-binding domain (RBD) is a major target for the development of diagnostics, vaccines and therapeutics directed against COVID-19. Important efforts have been dedicated to the rapid and efficient production of recombinant RBD proteins for clinical and diagnostic applications. One of the main challenges is the ongoing emergence of SARS-CoV-2 variants that carry mutations within the RBD, resulting in the constant need to design and optimise the production of new recombinant protein variants. We describe here the impact of naturally occurring RBD mutations on the secretion of a recombinant Fc-tagged RBD protein expressed in HEK 293 cells. We show that mutation E484K of the B.1.351 variant interferes with the proper disulphide bond formation and folding of the recombinant protein, resulting in its retention into the endoplasmic reticulum (ER) and reduced protein secretion. Accumulation of the recombinant B.1.351 RBD-Fc fusion protein in the ER correlated with the upregulation of endogenous ER chaperones, suggestive of the unfolded protein response (UPR). Overexpression of the chaperone and protein disulphide isomerase PDIA2 further impaired protein secretion by altering disulphide bond formation and increasing ER retention. This work contributes to a better understanding of the challenges faced in producing mutant RBD proteins and can assist in the design of optimisation protocols.

## 1. Introduction

Coronavirus disease 19 (COVID-19), an infectious disease caused by the severe acute respiratory syndrome coronavirus 2 (SARS-CoV-2), has been declared a global pandemic by the World Health Organization (WHO) in March 2020 [1]. About two years later, nearly 500 million confirmed cases and over 6 million deaths have been reported worldwide [1,2]. Tremendous efforts have been devoted to the development of diagnostic tools, vaccines and therapeutics to address this pandemic. Researchers have identified the receptor-binding domain (RBD) of the SARS-CoV-2 spike protein as a major target of neutralising antibodies, and thus as a protein domain of choice for the design and development of diagnostics, vaccine and therapeutic antibodies [3,4,5,6,7,8,9,10,11,12]. Notably, several studies reported the development and characterization of recombinant RBD proteins (amino acids 331-524 of the spike protein) fused to a C-terminal tag. These studies showed that recombinant RBD protein (amino acids 331-524) efficiently binds the angiotensin-converting enzyme 2 (ACE2) receptor, is efficiently recognised by neutralising antibodies from COVID-19 convalescent individuals, and elicits a strong neutralising antibody response when administered as an immunogen in BALB/c mice [13,14,15,16]. Of particular interest, Sun et al. expressed SARS-CoV-2 RBD (amino acids 331-524) either fused to a histidine (His) tag as a monomeric protein or fused to a human IgG1 Fc fragment to produce a Y-shaped dimeric protein. They showed that the dimeric RBD-Fc fusion protein bound the ACE2 receptor with an approximate 10-fold enhanced avidity compared to the monomeric RBD-His protein, suggesting an improved conformation of the dimeric RBD-Fc protein [16].

One of the main challenges of SARS-CoV-2 RBD protein engineering is the recurring emergence of SARS-CoV-2 variants that carry mutations within the RBD [17,18,19], resulting in the constant need to design and optimise the production of new recombinant protein variants. Typical complications that may occur during recombinant protein production include low yield, reduced stability, poor solubility and misfolding. Some of these production issues can be influenced by the amino acid sequence of the produced protein [20,21,22], which is of particular concern for the efficient production of mutated SARS-CoV-2 RBD recombinant proteins. To overcome these challenges, engineering and optimisation approaches have been proposed [23,24,25,26,27,28,29,30,31,32,33,34]. Additionally, mammalian cells have become a favoured system to produce recombinant proteins for clinical and diagnostic applications because they allow proper post-translational modifications and protein folding [35,36].

Nonetheless, protein misfolding remains a major limitation for the efficient production of secreted recombinant proteins in mammalian cells. During the maturation of extracellular proteins in the lumen of the endoplasmic reticulum (ER), protein folding is tightly coupled with the process of disulphide bond formation. Formation of native disulphide bonds and reduction of non-native erroneous disulphide bonds are essential steps assisting the proper folding of nascent polypeptides (co-translationally) and proteins (post-translationally) [37,38,39,40,41]. Disulphide bond formation, isomerisation and reduction are catalysed by protein disulphide isomerases (PDI), such as PDIA2 [42,43,44]. PDIA2 presents, in addition, a chaperone activity and is part of a multiprotein complex, together with classical molecular chaperones such as HSP90B1 (GRP94) or HSPA5 (GRP78 or BiP), that bind nascent proteins in the ER to promote efficient folding and prevent aggregations of proteins [37,45]. In addition to assisting protein folding, these chaperone-containing complexes can bind to unfolded and misfolded proteins accumulating and aggregating in the ER, thereby activating several ER homeostasis pathways [37,42,44,45,46,47,48]. Among them, the unfolded protein response (UPR) is an ER-stress response leading notably to the upregulation of chaperones such as PDIA2 or HSPA5, to enhance protein folding. In addition, protein degradation pathways are activated, including ER-associated protein degradation (ERAD), leading to the retro-translocation of the misfolded proteins into the cytosol to target their degradation by the proteasome, and ER-phagy, leading to lysosome-mediated selective degradation of ER domains [23,37,42,44,49].

In its native form, the RBD domain of the spike glycoprotein can take two conformational states: a closed state inaccessible to binding to the ACE2 receptor, and an open state accessible for ACE2 binding [50]. SARS-CoV-2 RBD contains four disulphide bonds (Cys336–Cys361, Cys379–Cys432, Cys391–Cys525, Cys480–Cys488) [51,52] that play a critical role in the structure and function of the RBD, by controlling the flexibility of the surface loops interacting with ACE2 [53]. SARS-CoV-2 RBD mutations, notably E484K, have been shown to modify RBD conformation [54,55,56]. Mutations within the RBD can affect its binding affinity to ACE2 or to neutralising antibodies, potentially impacting SARS-CoV-2 transmissibility and the response to vaccine or therapeutic monoclonal antibodies [54,55,56]. The impact of RBD mutations on its expression as a recombinant protein, on the other hand, has not been studied in detail. In this work, we describe the impact of RBD mutations present in the B.1.351 (beta) SARS-CoV-2 variant (K417N, E484K and N501Y) on the production of a recombinant RBD protein (amino acids 331-524) tagged with a C-terminal mouse IgG2a Fc fragment (thereafter named RBDmfc) in HEK-293F-derived cells, for diagnostic application. The production yield of the B.1.351 RBDmfc protein was strongly reduced compared to that of wild-type RBDmfc. The reduced yield of B.1.351 RBDmfc correlated with disulphide bond rearrangements and the partial retention of the mutant protein into the ER, possibly due to misfolding.

## 2. Materials and Methods

### 2.1. Protein Production and Purification

#### 2.1.1. Plasmid Constructs

**Figure 1:**
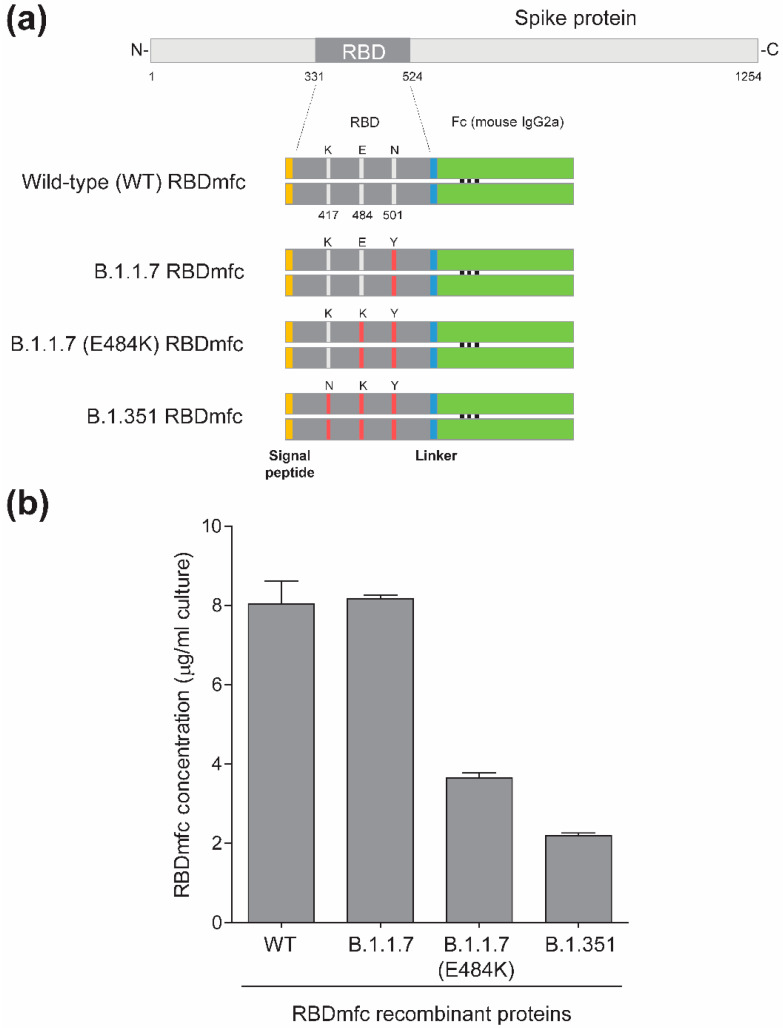
Expression of recombinant RBDmfc proteins in Expi293F cells. (**a**) Domain architecture of recombinant RBDmfc proteins investigated. The RBD domain (amino acids 331-524 of the spike protein) of SARS-CoV-2 isolate Wuhan-Hu-1 (wild type or WT) and variants B.1.1.7, B.1.1.7 (E484K) and B.1.351 was expressed by transient transfection into the Expi293F human cell line. RBD B.1.1.7 carries a single mutation (N501Y), RBD B.1.1.7 (E484K) two mutations (E484K and N501Y) and B.1.351 three mutations (K417N, E484K and N501Y) (shown in red). The respective RBD domains were expressed in fusion with an N-terminal optimised signal peptide to allow protein secretion and a C-terminal mouse IgG2a Fc fragment to facilitate purification on protein A affinity resin and to produce Y-shaped protein dimers. RBDmfc proteins are produced as dimers linked by disulphide bonds between their Fc fragments. One RBDmfc monomer is 437 amino acids long and has a predicted molecular weight of 49.2 kDa (upon cleavage of the signal peptide). (**b**) Concentration of RBDmfc proteins secreted in the culture medium of Expi293F cells, measured by Octet RED96 and expressed in μg/mL culture. Values are mean (standard deviation) of two independent experiments. RBD mutants carrying the E484K mutation were expressed at lower levels in the medium of Expi293F cells. The level of secreted B.1.351 RBDmfc was 27% that of WT RBDmfc. By contrast, mutation N501Y had no impact on the secreted protein production yield.

Eukaryotic expression vectors for wild-type (WT) and mutant RBDmfc were designed using codon optimisation, produced, assembled and their sequence confirmed by e-Zyvec (Loos, France). The empty vector is a proprietary plasmid containing the human cytomegalovirus (CMV) immediate early promoter for expression in mammalian cells and an ampicillin resistance gene for selection in *E. coli*. Expression plasmid for WT RBDmfc is composed of an N-terminal codon-optimised DNA sequence coding for the signal peptide of the SARS-CoV-2 spike protein (MFVFLVLLPLVSSQC), fused to the sequence encoding amino acids 331-524 of the SARS-CoV-2 spike protein (SARS-CoV-2 isolate Wuhan-Hu-1, GenBank: QHD43416.1), a sequence encoding a nine amino acid linker region, and a C-terminal mouse IgG2a Fc sequence, inserted into the empty CMV-driven expression vector. Plasmid constructs encoding mutant RBDmfc proteins were generated as the WT construct, except for nucleotide substitutions within the RBD domain at codons mutated in the respective SARS-CoV-2 variants (according to the GISAID database), as follows: B.1.1.7 RBDmfc (one amino acid mutation N501Y), B.1.1.7 (E484K) RBDmfc (two amino acid mutations E484K and N501Y), and B.1.351 RBDmfc (three amino acid mutations K417N, E484K and N501Y) (Figure 1a). One encoded RBDmfc monomer contains 437 amino acids and has a predicted molecular weight of 49.2 kDa upon signal peptide cleavage. The human PDIA2 cDNA (NM_006849.2) expression plasmid (SinoBiological, HG18027-UT) was purchased from Interchim (Montluçon, France).

#### 2.1.2. Plasmid Production and Purification

Plasmids were transformed into One Shot TOP10 Chemically Competent *E. coli* (ThermoFisher Scientific, Carlsbad, CA, USA; C404010) with the rapid chemical transformation procedure proposed by the manufacturer. Transformed *E. coli* were spread on LB Agar (BD, Sparks, NV, USA; 244520) supplemented with 100 µg/mL ampicillin (Roche/Sigma-Aldrich/Merck, Darmstadt, Germany; 10835242001), and incubated at 37 °C overnight. Isolated colonies were grown overnight at 37 °C in LB medium (BD, Sparks, NV, USA; 244620) containing 100 µg/mL ampicillin. *E. coli* cultures were centrifuged for 30 min at 5000× *g* and bacterial pellets were conserved at −20 °C until plasmid extraction. Pellets were processed with ZymoPURE II Plasmid Maxiprep Kit (Zymo Research, Irvine, CA, USA; D4203) using the standard protocol with the EndoZero Spin Columns (Zymo Research, Irvine, CA, USA; C1051-10). Purified plasmid concentration was measured on NanoDrop One C (ThermoFisher Scientific, Carlsbad, CA, USA) at 260 nm. Purified plasmids were stored at −20 °C until transfection.

#### 2.1.3. Plasmid Transient Transfection and Protein Production in Expi293F Cells

Plasmid constructs were transiently transfected into Expi293F cells (A14528), using Expi293 Expression Medium (A14351-01), ExpiFectamine 293 Transfection Kit (A14526) and Opti-MEM I Reduced Serum Medium (31985-047) following the Expi293 Expression System transfection protocol (all from ThermoFisher Scientific, Carlsbad, CA, USA). Expi293F cells are human embryonic kidney (HEK) cells derived from the originally described HEK-293 cell line [57] that has been adapted to suspension growth (293F). Cell number was determined using a NucleoCounter NC-200 automated cell counter (ChemoMetec, Allerod, Denmark; 900-0200) and the Via1-Cassette (ChemoMetec, Allerod, Denmark; 941-0012).

For transient transfections, 1 µg total plasmid DNA was used per ml cell suspension. Accordingly, transfection experiments with 100% RBDmfc-expressing plasmid used 1 µg RBDmfc plasmid DNA (per ml cell suspension), while experiments with 95% and 70% RBDmfc-expressing plasmid used 0.95 and 0.7 µg RBDmfc plasmid DNA (per ml cell suspension), complemented with 0.05 (5%) and 0.3 (30%) µg, respectively, of either empty or PDIA2-expressing plasmid. Transfected cells were incubated for four days in a Multitron incubator (INFORS HT, Bottmingen, Switzerland) at 37 °C, 8% CO_2_, under shaking at 125 rpm with a 19 mm orbital.

#### 2.1.4. Secreted and Intracellular Protein Fraction Preparation

Transfected cells were enumerated and harvested four days after transfection and cell suspensions were centrifuged for 15 min at 5000× *g*. Protein-containing medium supernatants (secreted RBDmfc protein fraction) were filtered on 0.22 µm PVDF membrane (Millipore/Merck, Darmstadt, Germany; S2GVU05RE) and stored at 4 °C. Cell pellets were stored at −20 °C until cell lysate preparation.

Frozen cell pellets corresponding to approximately 5.4 × 10^7^ cells in 8 mL cell suspension were suspended by pipetting in 1 mL phosphate-buffered saline (PBS) pH 7.2 (BioMérieux, Marcy l’étoile, France; 75511) containing 20 mM Octyl β-D-glucopyranoside (Sigma-Aldrich/Merck, Darmstadt, Germany; O8001) and 5 units/mL Benzonase Nuclease (Merck, Darmstadt, Germany; 70664). After 10 min lysis at 4 °C, lysates were centrifuged for 10 min at 10,000× *g* and cellular extract supernatants (intracellular RBDmfc protein fraction) were stored at 4 °C until analysis.

#### 2.1.5. RBDmfc Protein Concentration Determination

Measurement of RBDmfc protein concentrations in cell media (secreted protein fraction) and cell lysates (intracellular protein fraction) was performed on an Octet RED96 (Sartorius, Göttingen, Germany) using Octet Protein A (ProA) Biosensors (Sartorius, Göttingen, Germany; 18-501). Calibration curves were made in duplicate using 200 µL purified WT RBDmfc proteins diluted in 1× Octet Kinetics Buffer (Sartorius, Göttingen, Germany; 18-1105; 10× buffer diluted 1:10 in PBS) to a final concentration of 0, 3.125, 6.25, 12.5, 25.0 and 50 µg/mL, respectively. Samples (200 µL) were measured in duplicate in a 96-well microplate (Greiner Bio-One, Frickenhausen, Germany; 655209) either undiluted (cell supernatants) or diluted 1:4 in 1× Kinetics Buffer (cell pellet extracts). Octet ProA biosensor tips were dipped in the samples for a 120-sec measurement. Between each measurement, ProA biosensors were regenerated with three cycles dipping in 10 mM glycine (Merck, Darmstadt, Germany; 104201.250) pH 1.7 and neutralised in 1× Kinetics Buffer.

#### 2.1.6. RBDmfc Purification and Quantification

Cell media and cell extract supernatants containing RBDmfc proteins were purified using a MabSelect protein A resin (GE Healthcare, Uppsala, Sweden; 17819902) on an AKTA FPLC Basic 10 (GE Healthcare, Uppsala, Sweden). Resin was equilibrated in PBS pH 7.2, supernatants or cellular extracts were loaded onto the resin and washed with 20 column volumes (CV) PBS pH 7.2. RBDmfc proteins bound to the resin were eluted with 10 CV of a proprietary acidic buffer. Eluted fractions were concentrated on Vivaspin 20 (10,000 molecular weight [MW] cutoff filtration units; Sartorius, Göttingen, Germany; VS2002) and dialysed against PBS pH 7.2 using the same Vivaspin 20 filtration unit.

Concentration of the purified RBDmfc proteins was measured with a NanoDrop One C spectrophotometer (ThermoFisher Scientific, Carlsbad, CA, USA) at 280 nm. Protein concentrations were calculated using a UV extinction coefficient ε (0.1%, 280 nm) of 1.357 for WT RBDmfc protein and 1.386 for B.1.351 RBDmfc protein. UV extinction coefficients were determined by computation using the ExPASy ProtParam tool (https://web.expasy.org/protparam, accessed on 23 August 2022), according to the amino acid composition of the respective proteins and assuming that all pairs of cysteine residues form cystines (i.e., are joined by a disulphide bond).

### 2.2. Protein Analysis by SDS-PAGE and Western Blotting

#### 2.2.1. SDS-PAGE

For secreted and intracellular protein fraction analysis by sodium dodecyl sulphate–polyacrylamide gel electrophoresis (SDS-PAGE), samples were mixed 1:4 in NuPAGE LDS Sample Buffer (4×) (ThermoFisher Scientific, Carlsbad, CA, USA; NP0007), heated 10 min at 80 °C and centrifuged 1 min at 14,000× *g*. A volume of 18 µL denatured sample and of 10 µL Precision Plus Protein Kaleidoscope Prestained Protein Standards (Bio-Rad Laboratories, Hercules, CA, USA; 1610375) were loaded on NuPAGE 4–12% Bis-Tris Gels (ThermoFisher Scientific, NP0321), and electrophoresis was conducted in 1× NuPAGE MOPS SDS Running Buffer (ThermoFisher Scientific, NP0001) at constant voltage (180 V) for 1 h.

For purified RBDmfc protein analysis by SDS-PAGE, protein samples (0.1 μg/mL in PBS) were mixed 1:4 in NuPAGE LDS Sample Buffer (4×), and 10 µL denatured and heated samples were loaded and separated on SDS-PAGE, as above.

Coomassie staining of proteins separated by SDS-PAGE was performed with the GelCode Blue Safe Protein Stain (ThermoFisher Scientific, 24596) following the manufacturer’s standard protocol. Silver staining of proteins separated by SDS-PAGE was performed with the SilverQuest Silver Staining Kit (ThermoFisher Scientific, LC6070) using the manufacturer’s Fast Staining protocol.

#### 2.2.2. Western Blotting

Protein transfer for western blotting was done with the Trans-Blot Turbo Mini 0.2 μm Nitrocellulose Transfer Packs (Bio-Rad Laboratories, Hercules, CA, USA; 1704158) using a Trans-Blot Turbo Transfer System (Bio-Rad Laboratories) with its pre-programmed 1.5 MM GEL protocol.

Following protein transfer, membranes were blocked for 1 h under orbital agitation in PBS, 10% dry skimmed milk (Régilait, Mâcon, France) (for all antibodies except human sera) or in PBS, 1% bovine serum albumin (BSA) (Proliant Biologicals, Ankeny, IA, USA; 68100) (for human sera). Blocked membranes were incubated for 2 h with the following primary antibodies in 10 mL PBS: Alkaline phosphatase-conjugated anti-RBD (proprietary pool of ten mouse monoclonal antibodies generated using wild-type RBD as immunogen; 0.5 µg/mL); Alkaline Phosphatase-AffiniPure Sheep Anti-Mouse IgG (H+L) (Jackson ImmunoResearch Laboratories, West Grove, OK, USA; 515-055-062; 0.5 µg/mL) for the detection of the mouse Fc region of the RBDmfc proteins (anti-Fc blotting); rabbit polyclonal anti-PDIA2 (ThermoFisher Scientific, PA5-112644; 0.5 µg/mL); mouse monoclonal anti-HSPA5 (GRP78, clone 1H11-1H7) (ThermoFisher Scientific, MA5-27686; 1 µg/mL); mouse monoclonal anti-βactin (clone AC-15) (ThermoFisher Scientific, AM4302; 0.2 µg/mL); rat monoclonal anti-HSP90B1 (GRP94, clone 9G10) (ThermoFisher Scientific, MA3-016; 1:1000); COVID-19 patient antisera (1:100). COVID-19 patient antisera were defined as follows: the WT SARS-CoV-2 serum (Etablissement Français du Sang [EFS], France) was from a COVID-19 patient of the pre-B.1.351 variant era, tested positive for SARS-CoV-2 IgG (and negative for SARS-CoV-2 IgM) using commercial serology assays (SARS-CoV-2 IgG and IgM ELISA, Euroimmun, Lübeck, Germany; SARS-CoV-2 IgG and IgM VIDAS assays, bioMérieux, Craponne, France); the B.1.351 SARS-CoV-2 serum (Centre Hospitalier Saint Joseph Saint Luc, Lyon, France) was collected from a COVID-19 patient 40 days after being tested positive for B.1.351 SARS-CoV-2 by RT-PCR (thus expected to be SARS-CoV-2 IgG-positive). It is worth noting that the anti-RBD monoclonal antibody pool raised against WT RBD also recognises B.1.351 RBD, albeit less efficiently, thus generating weaker signals by western blot for the same amount of target protein. Moreover, these antibodies being generated against matured (glycosylated) WT RBD, they recognise intracellular (not fully glycosylated) RBD proteins very weakly (data not shown).

Following incubation with primary antibodies, membranes were washed seven times with 20 mL PBS, 0.05% Tween 20 (Sigma-Aldrich/Merck, Darmstadt, Germany; 8.22184.0500). Western blots using alkaline phosphatase-conjugated primary antibodies (anti-RBD, anti-mFc) were directly submitted to alkaline phosphatase activity detection. Western blots using non-conjugated primary antibodies were incubated for 2 h with the following secondary antibodies in 10 mL PBS: Alkaline Phosphatase-AffiniPure Goat Anti-Rabbit IgG (H+L) (Jackson ImmunoResearch Laboratories, 111-055-144; 0.3 µg/mL) for anti-PDIA2 blots; Alkaline Phosphatase-AffiniPure Goat Anti-Mouse IgG F(ab’)2 Fragment Specific (Jackson ImmunoResearch Laboratories, 115-055-072; 0.7 µg/mL) for anti-HSPA5, anti-βactin and anti-HSP90B1 blots; alkaline phosphatase mouse anti-human IgG (proprietary; 1 µg/mL) for blots using anti-SARS-CoV-2 human sera. Membranes incubated with secondary antibodies were washed seven times with 20 mL PBS, 0.05% Tween 20.

Alkaline phosphatase activity detection was performed by incubating membranes with 7 mL 1-Step NBT/BCIP Substrate Solution (ThermoFisher Scientific, 34042) until desired signal intensity and blocked by three washes in 100 mL demineralised water.

#### 2.2.3. Image Acquisition

Image acquisition was performed using the ChemiDoc XRS+ Gel Imaging System (Bio-Rad Laboratories, Hercules, CA, USA) and the Image Lab Software (Bio-Rad Laboratories). The Image Lab software was set to the colorimetric gel acquisition mode for Coomassie-stained gels, the silver stain mode for silver-stained gels, and the colorimetric blot acquisition mode for western blots. The signal intensity of western blot bands was quantified, and the relative signal was determined using the Image Lab Software (Bio-Rad Laboratories). Uncropped SDS-PAGE images are shown in Appendix A.

### 2.3. Protein Analysis by Enzyme-Linked Immunosorbent Assay (ELISA)

Purified secreted WT and B.1.351 RBDmfc proteins (100 µL at 5 µg/mL) were coated onto wells of a 96-well microplate (Greiner Bio-One, Frickenhausen, Germany; 655001) overnight at room temperature. Microplate wells were washed three times with 300 µL PBS, 0.05% Tween 20 and blocked for 3 h at room temperature with 200 µL PBS, 1% BSA (Proliant Biologicals, Ankeny, IA, USA; 68100). Wells were washed three times as before and incubated for 2 h at room temperature with 100 µL of the respective human sera (1:200 in PBS) or controls, as follows: the WT SARS-CoV-2 serum (same as that used in western blot) was added to duplicate wells coated with purified WT RBDmfc, the B.1.351 SARS-CoV-2 serum (same as that used in western blot) was added to duplicate wells coated with purified B.1.351 RBDmfc, a pre-pandemic human serum (EFS, Saint-Denis, France) used as a negative control was added to duplicate WT- and B.1.351 RBDmfc-coated wells, and PBS (blank) was added to four WT- and B.1.351 RBDmfc-coated wells. Microplate wells were washed three times as above and RBD-specific SARS-CoV-2 IgG were detected by incubation for 2 h at room temperature with 100 µL of a proprietary alkaline phosphatase mouse anti-human IgG (0.16 µg/mL in PBS, 0.05% Tween 20). Microplate wells were washed five times with 300 µL PBS, 0.05% Tween 20 and specific immunocomplexes were revealed by incubating for 20 min at 37 °C in 100 µL 4-Nitrophenyl phosphate disodium salt hexahydrate (PNPP; Sigma-Aldrich/Merck, Darmstadt, Germany; P5994) at 1 mg/mL in a proprietary diethanolamine (DEA)-HCl buffer. Coloration was stopped by adding 100 µL 1N NaOH (VWR, Radnor, PA, USA; 35256-1L). Absorbance was measured at 405–490 nm on a BioTek ELX808 microplate reader (Agilent, Santa Clara, CA, USA) and data were analysed using the BioTek Gen5 Microplate Reader and Imager Software 3.10 (Agilent).

### 2.4. Protein Reduction and Deglycosylation Experiments

For disulphide bond reduction of RBDmfc proteins secreted in cell media, 26 µL purified RBDmfc proteins at 300 µg/mL in PBS were mixed with 4 µL NuPAGE Sample Reducing Agent (10×) (ThermoFisher Scientific, NP0009; containing 500 mM Dithiothreitol [DTT]) or 4 µL of PBS for the non-reduced condition, and 10 µL NuPAGE LDS Sample Buffer (4×) (ThermoFisher Scientific, NP0007). Then, 20 µL reaction mixes were heated for 10 min at 80 °C and loaded on SDS-PAGE. Separated proteins were analysed by Coomassie staining, as described above.

For Peptide-*N*-Glycosidase F (PNGase) digestion, 20 µL purified RBDmfc proteins (secreted and intracellular protein fractions) at 120 µg/mL in PBS, were incubated with 4 µL PNGase F (10 U/µL; Promega, V483A) and 16 µL 50 mM ammonium bicarbonate buffer (Merck, 09830). For the non-digested control, PNGase F was replaced by 4 µL 50 mM ammonium bicarbonate buffer. For endoglycosidase H (EndoH) digestion, 20 µL purified RBDmfc proteins (secreted and intracellular protein fractions) at 120 µg/mL in PBS, were incubated with 4 µL EndoH (500 U/µL; Promega, V487A), 4 µL 10× EndoH Reaction Buffer (Promega, Madison, WI, USA; V490A) and 12 µL Milli-Q water. For the non-digested control, EndoH was replaced by 4 µL Milli-Q water. PNGase and EndoH reactions were incubated for 16 h at 37 °C and 20 µL were loaded on SDS-PAGE, followed by Coomassie staining, as described above.

### 2.5. Hydrophobicity Determination by HIC-MALS

Hydrophobic interaction chromatography hyphenated to multi-angle light scattering detection (HIC-MALS) measurements were performed using an UltiMate 3000 HPLC and UHPLC System (ThermoFisher Scientific) equipped with a quaternary RS pump, a WPS-3000FC thermostated sampler and fraction collector, and a DAD-3000RS Diode Array Detector set at 280 nm. Multi-angle light scattering data were acquired from a Dawn 8+ MALS detector and interpreted with the Astra 7 software (version 7.3.2.19; Wyatt Technology, Santa Barbara, CA, USA). RBDmfc sample molar mass calculations were performed using a UV extinction coefficient ε (0.1%, 280 nm) of 1.357 for WT RBDmfc protein and 1.386 for B.1.351 RBDmfc protein, as described above (Section 2.1.6).

Analytical HIC measurements of the RBDmfc samples were performed using an AdvanceBio HIC column (4.6 mm inner diameter × 100 mm length, 3.5 µm particle size; Agilent). The autosampler and fraction collector was set at 5 °C, 10 µg protein sample was injected, and the mobile-phase flow rate was set at 0.5 mL/min. A 20-min linear gradient was applied from 100% 2 M ammonium sulphate containing 0.05 M phosphate buffer pH 7.0 to 100% 0.05 M phosphate buffer pH 7.0, followed by a 5 min washing step with 100% 0.05 M phosphate buffer pH 7.0 to assure that all bound impurities were removed. The column was then re-equilibrated at starting mobile phase conditions using a 5 min gradient and 10 min step. Fraction collection started at 23 min and ended at 32 min, using a collection period of 30 s. Collected fractions were stored in a Nunc 2.0 mL U-bottom Polypropylene DeepWell 96-well microplate (ThermoFisher Scientific, 278752) within the thermostated collector until the end of HIC separation, then immediately loaded on SDS-PAGE for silver staining analysis, as described above.

### 2.6. Intact Mass Analysis by MALDI-TOF

Intact mass spectrometry was performed by matrix-assisted laser desorption ionization coupled to time-of-flight (MALDI-TOF) using an Axima Confidence instrument with Launchpad 2.9.4.1 software (Shimadzu, Kyoto, Japan). Spectra analysis was performed with mMass 5.5.0 (Martin Strohalm, under GNU license). Purified WT and B.1.351 RBDmfc protein samples (20 µg) were used untreated or thioreduced with 50 mM DTT. A sample volume of 10 µL was desalted and concentrated with ZipTip C4 resin (Merck, ZTC04S960) then eluted with 3 µL saturated α-Cyano-4-hydroxycinnamic acid (HCCA) matrix (Sigma-Aldrich/Merck, C2020) in 60% acetonitrile (ACN), 0.1% trifluoroacetic acid (TFA) on a 384-well stainless-steel target plate (Shimadzu, DE1580TA). Instrument calibration was performed with BSA (Sigma-Aldrich/Merck, A8654), using four points [2×BSA]+, [BSA]+, [BSA]2+, [BSA]3+ with the following settings: laser power of 82, 1000 profiles accumulation, and raster enabled. All samples were acquired with the same tuning. After acquisition, all averaged spectra profiles were converted to American Standard Code for Information Interchange (ASCII) format and imported into the mMass software, where only Gaussian smoothing of 500 *m*/*z* (mass-to-charge ratio) was applied as peak processing.

### 2.7. Size-Exclusion Chromatography

Size-exclusion chromatography (SEC) is a relative method allowing estimation of the apparent MW of proteins in solution, based on the retention time. Analytical SEC was performed on an H-Class Acquity Ultra Performance Liquid Chromatography system (UPLC, Waters, Milford, CT, USA). Briefly, the SEC column (Waters, BEH200) was preequilibrated in PBS 3× pH 6.0 for 30 min at 0.4 mL/min, as described by the manufacturer. Purified WT and B.1.351 RBDmfc protein samples were filtered (Ultrafree MC-GV Durapore PVDF 0.22 μm), and a sample volume of 10 µL was injected into the column at 30 °C at a flow rate of 0.4 mL/min. UV absorbance of the eluate was monitored at a wavelength of 280 nm.

### 2.8. Thermal Stability Determination by Differential Scanning Calorimetry

Differential scanning calorimetry (DSC) was performed using a CSC-6300 NanoDSC III calorimeter, and data were acquired and interpreted using the DSCRun 4.4.9 and NanoAnalyze 3.7.5 software, respectively (TA Instruments, New Castle, DE, USA). Purified, dialysed RBDmfc protein samples were adjusted to 0.605 mg/mL with PBS. The instrument sample cell was filled with the protein sample (exactly 299 µL), and the reference cell was filled with PBS (the same PBS batch as used for sample dialysis and concentration adjustment). A PBS blank was also performed for further baseline correction.

Data were collected using a heating rate of 1 °C/min from 20 °C to 100 °C under 3 atmospheric pressure and after a pre-equilibration step of 600 sec at 20 °C. The sample’s raw thermogram (µJ/s vs. temperature) was baseline subtracted versus the PBS thermogram, then converted to molar heat capacity (defined as the amount of energy in the form of heat needed to raise the temperature of one mole of purified RBDmfc protein by one Kelvin) using their respective molecular weight to obtain kJ/mol.K vs. temperature thermograms. The integration of transition peaks was then achieved using a sigmoidal baseline method before calculating thermodynamic parameters.

## 3. Results

### 3.1. Impact of RBD Mutations on the Production Yield of Recombinant RBDmfc Fusion Proteins in 293F Cells

The RBD domain (amino acids 331-524) of SARS-CoV-2 isolate Wuhan-Hu-1 (hereafter referred to as wild type or WT) and variants B.1.1.7, B.1.1.7 (E484K) and B.1.351 was expressed by transient transfection into the Expi293F human cell line. RBD B.1.1.7 carries a single mutation (N501Y), RBD B.1.1.7 (E484K) carries two mutations (E484K and N501Y) and B.1.351 carries three mutations (K417N, E484K and N501Y) (Figure 1a). The respective RBD domains were expressed as fusion proteins with an N-terminal optimised signal peptide to direct newly synthesised proteins toward the secretory pathway and a C-terminal mouse IgG2a Fc fragment to facilitate the purification by protein A- affinity resin of the recombinant proteins (hereafter referred to as RBDmfc proteins). RBDmfc proteins are expected to be produced as dimers linked by disulphide bonds between their Fc fragments (Figure 1a). The concentration of secreted proteins in the medium of Expi293F-expressing cells, measured by Octet RED96, differed between WT and some of the mutant RBDmfc proteins (Figure 1b). Although WT and B.1.1.7 variant proteins were produced in comparable amounts in cell supernatants (around 8 μg/mL culture), RBD mutants B.1.1.7 (E484K) and B.1.351 showed a reduced yield, with B.1.351 RBDmfc revealing the lowest concentration (about 4-times lower than WT) (Figure 1b). These observations indicate that the single RBD mutation N501Y had no impact on protein yield while mutation E484K had a strong impact, further enhanced by the presence of the K417N mutation.

To identify the cause of this difference in production level, we focused on B.1.351 RBDmfc, which showed the lowest secretion level, and further analysed its expression characteristics and biochemical properties, in comparison to WT RBDmfc.

### 3.2. Disulfide Bonds and Functionality of WT and B.1.351 RBDmfc Proteins Produced in 293F Cells

**Figure 2:**
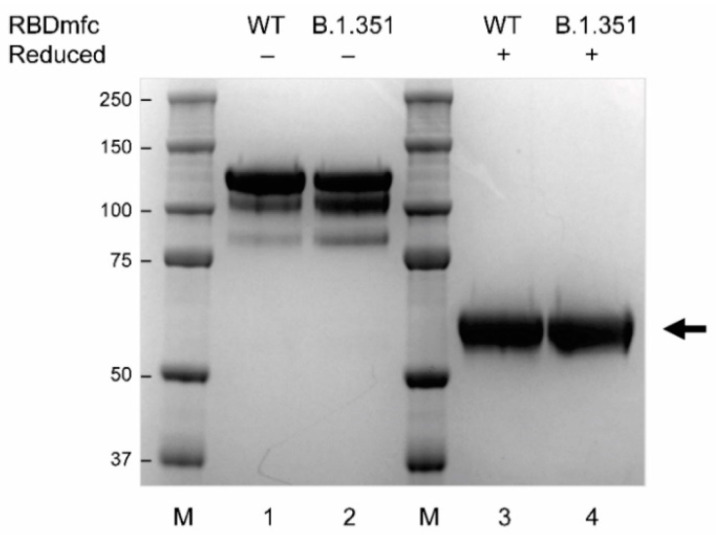
Secreted WT and B.1.351 RBDmfc proteins exhibit different disulphide bond rearrangements. RBDmfc proteins released into the culture medium of transfected Expi293F cells were purified. Reduced and non-reduced fractions were analysed by SDS-PAGE and Coomassie staining. Protein ladder (M) was run in parallel (apparent MW in kDa indicated on the left). Three discrete bands of approximately 120, 105 and 85 kDa apparent MW were detected for the non-reduced fractions (lanes 1 and 2), resolving into one single band of approximatively 59 kDa upon reduction (lanes 3 and 4, black arrow). Quantification of the signal intensity of the three non-reduced WT and B.1.351 bands revealed different proportions of the upper to lower bands between WT (66.0%, 22.8% and 11.2%) and B.1.351 (51.2%, 32.9% and 15.9%) RBDmfc proteins, respectively. Uncropped SDS-PAGE image is shown in Appendix A Figure A1.

Purified, secreted WT and B.1.351 RBDmfc proteins were separated by SDS-PAGE and stained with Coomassie (Figure 2, lanes 1 and 2). WT and B.1.351 RBDmfc proteins were detected as three bands of approximately 120, 105 and 85 kDa apparent molecular weight (MW). The respective intensity of these three bands differed between WT and B.1.351 RBDmfc, the upper MW band being predominant but more abundant in WT than in B.1.351 RBDmfc preparations (66.0% vs. 51.2%), whereas the two lower MW bands being more abundant in B.1.351 than in WT RBDmfc preparations (32.9% vs. 22.8% for the ~105 kDa band, and 15.9% vs. 11.2% for the ~85 kDa band) (Figure 2). Upon reduction in the presence of 50 mM DTT, these three bands resolved into one single band of approximately 59 kDa (Figure 2, lanes 3 and 4). This observation confirms that the three non-reduced bands correspond to RBDmfc dimers bounds by disulphide bonds between their Fc segments, as expected from their size and structure (Figure 1a), converted into RBDmfc monomers upon cleavage of the disulphide bonds under reducing conditions. The apparent MW of 59 kDa of the reduced protein species (compared to the predicted size of 49 kDa for a non-glycosylated monomer) indicates that the secreted protein is fully glycosylated. It also suggests that WT and B.1.351 RBDmfc proteins differ in their disulphide bond arrangements, resulting in three secreted forms produced in different proportions and running at distinct apparent MW by SDS-PAGE.

Because differences in disulphide bond arrangements might impact protein function, the functionality of the purified secreted WT and B.1.351 RBDmfc recombinant proteins was verified. RBDmfc proteins were developed and produced for use in immunoassays. Accordingly, their activity was evaluated as to their ability to be recognised by SARS-CoV-2-specific IgG under both denaturing (western blot) and non-denaturing (enzyme-linked immunosorbent assay [ELISA]) conditions (Figure S1). Western blot analyses demonstrated that sera from patients infected with SARS-CoV-2 (Wuhan isolate [WT] or B.1.351 variant) recognised the three protein species (of WT and B.1.351 RBDmfc, respectively), with a stronger detection of the upper MW band (Figure S1a). Similarly, ELISA performed on coated RBDmfc proteins (WT or B.1.351) using COVID-19 patient sera, demonstrated strong antigen-antibody (IgG) recognition signals (about 100-fold over negative serum background) for both RBDmfc proteins (Figure S1b). These experiments thus demonstrate that both WT and B.1.351 RBDmfc recombinant proteins are reactive toward SARS-CoV-2-specific IgG from patient sera.

### 3.3. Impact of PDIA2 Expression on the Production and Secretion of WT and B.1.351 RBDmfc Fusion Proteins in 293F Cells

**Figure 3:**
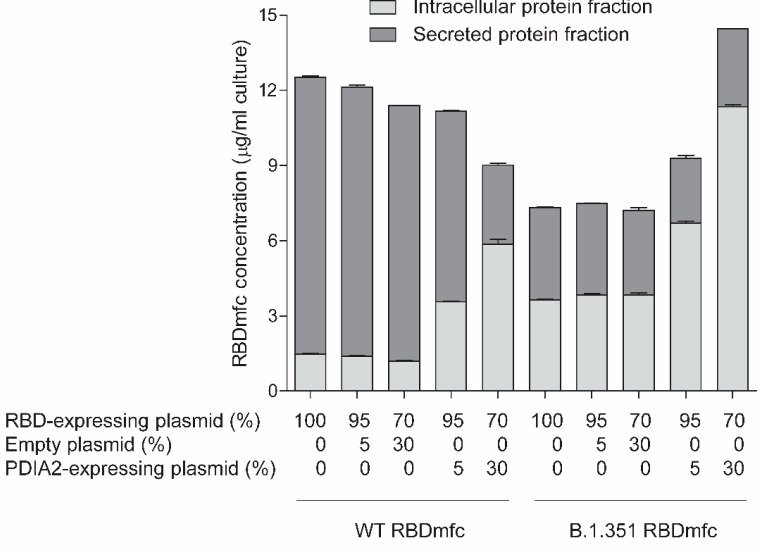
Concentration of intracellular and secreted recombinant RBDmfc proteins upon co-transfection of Expi293F cells with PDIA2-expressing or empty vectors. Expi293F cells were transfected with 1 µg plasmid DNA per ml cell suspension (“100%” thus corresponding to 1 µg plasmid DNA). In co-transfection experiments, the respective proportion of plasmid expressing RBDmfc, PDIA2 or empty plasmid (to complete to 1 µg plasmid DNA) is indicated below each bar. Accordingly, 100%, 95%, 70% or 5% plasmid DNA indicates that 1, 0.95, 0.7 or 0.05 µg plasmid DNA (per ml cell suspension) was used for transfection, respectively. Intracellular and secreted protein fractions were prepared four days after transfection. Protein concentration was measured by Octet RED96 and expressed in μg/mL culture. Values are mean (standard deviation) of duplicate Octet RED96 measurements of one representative experiment (out of two independent experiments).

The concentration of intracellular and secreted WT and B.1.351 RBDmfc proteins was then evaluated upon expression in Expi293F cells. The intracellular concentration of WT RBDmfc was low while its concentration in the culture medium was high (Figure 3, 100% WT RBDmfc condition), demonstrating that WT RBDmfc is efficiently secreted. By contrast, the level of secreted B.1.351 RBDmfc was low, as described above (Figure 1b), while its intracellular concentration was higher compared to that of WT RBDmfc-expressing cells (Figure 3, 100% B.1.351 RBDmfc condition). This suggests that a pool of B.1.351 RBDmfc proteins is retained inside the cells and that the lower yield of protein secretion is likely due to impaired trafficking rather than impaired translation. These observations also confirm that WT and B.1.351 RBDmfc secreted proteins are processed differently via disulphide bond formation and isomerisation during maturation in the ER. Disulphide bond formation and isomerisation being influenced by protein folding (and vice-versa), these results in turn suggest that WT and B.1.351 RBDmfc proteins might differ in their folding, resulting in impaired processing of B.1.351 RBDmfc along the secretory pathway, possibly explaining its reduced yield compared to WT RBDmfc upon expression in Expi293F cells.

Previous studies have shown that overexpression of chaperones, including protein disulphide isomerase PDIA2, can reduce the amount of aggregated misfolded proteins and promote protein refolding and secretion [46,47,48]. To address the possible implication of protein misfolding and disulphide bond rearrangements in the impaired secretion of B.1.351 RBDmfc, we tested the effect of overexpression of the protein disulphide isomerase and chaperone PDIA2 on B.1.351 RBDmfc protein expression and secretion in Expi293F cells.

Overexpression of PDIA2 led to a dose-dependent increase of the intracellular concentration of both WT and B.1.351 RBDmfc proteins (Figure 3). This increase in intracellular RBDmfc was accompanied by a strong decrease in the concentration of secreted WT RBDmfc, while the level of secreted B.1.351 RBDmfc remained low (Figure 3). This observation indicates that overexpression of PDIA2 interfered with the processing and secretion of WT RBDmfc, possibly by altering disulphide bond formation and isomerisation, while it did not improve secretion of B.1.351 RBDmfc. In addition, the strong accumulation of intracellular B.1.351 RBDmfc upon overexpression of PDIA2 suggests a possible protection and stabilisation of the RBD protein by PDIA2, but no promotion of folding and secretion, as anticipated. These results also confirm that the low yield of secreted B.1.351 RBDmfc was not due to impaired translation but occurred post-translationally.

On the whole, these results demonstrate that WT and mutant RBDmfc proteins are differentially processed along the secretory pathway and suggest possible differences in protein folding and disulphide bond arrangements during maturation in the ER.

**Figure 4:**
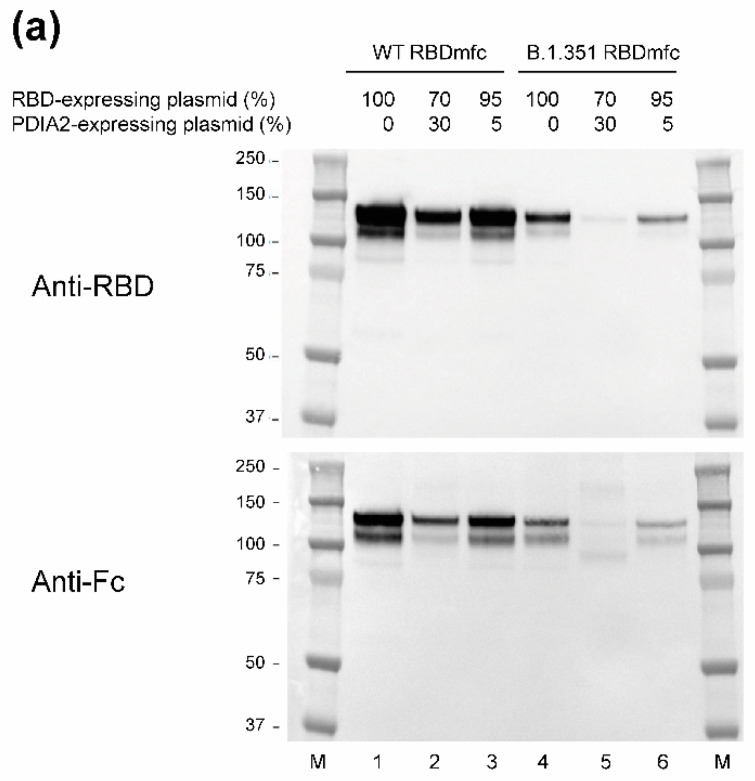

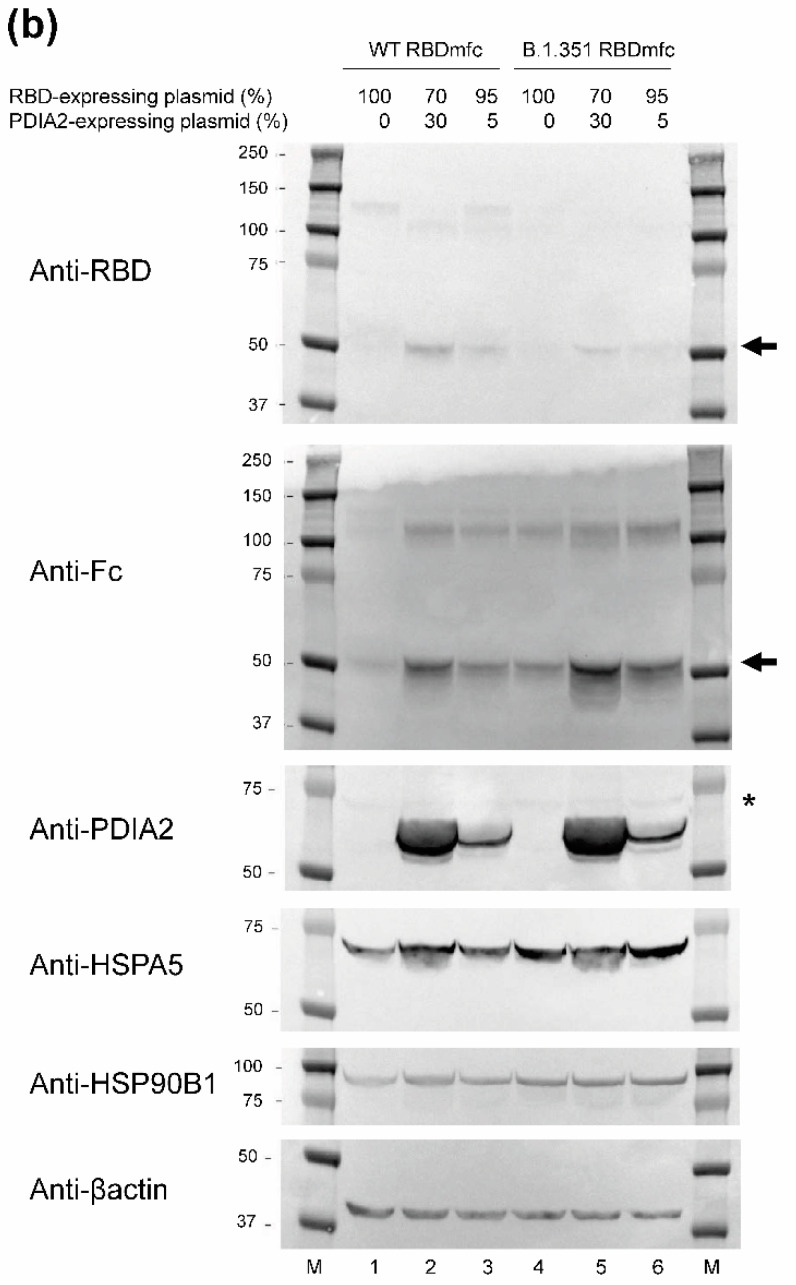
Western blots of secreted (**a**) and intracellular (**b**) protein fractions of Expi293F cells expressing WT and B.1.351 RBDmfc together with increasing amounts of PDIA2. Secreted and intracellular protein fractions described in Figure 3 were analysed by Western blot using antibodies directed against the recombinant RBDmfc proteins (anti-RBD, anti-Fc), against chaperones (anti-PDIA2, anti-HSPA5, anti-HSP90B1), and against βactin as loading control. M, protein ladder (apparent MW in kDa indicated on the left); *, endogenous PDIA2 protein (anti-PDIA2 blot); arrow, RBDmfc proteins (anti-RBD and anti-Fc blots in panel (**b**)) likely lacking disulphide bonds and mature glycosylations, and thus running in SDS-PAGE with an apparent MW of 50 kDa (compare to 59 kDa reduced, mature proteins shown in Figure 2, lanes 3 and 4). Quantification of signal intensity of chaperones is shown in Figure S2. Data are from one representative experiment (out of three independent experiments). Uncropped blot images are shown in Appendix A Figure A3 and Figure A4.

Western blot analysis of the intracellular and secreted protein fractions shown in Figure 3 demonstrated PDIA2 overexpression in cells co-transfected with increasing amounts of the PDIA2-expressing plasmid (Figure 4b, blot anti-PDIA2; lanes 2, 3, 5, 6), therefore confirming the dose-dependent effect of PDIA2 on the intracellular accumulation of RBDmfc (Figure 3). Anti-RBD and anti-Fc blots on the secreted protein fractions (Figure 4a) confirmed that intracellular accumulation of RBDmfc induced by overexpressed PDIA2 (Figure 3) correlated with a diminished secretion of both WT and B.1.351 proteins (Figure 4a, lanes 2 and 5).

An anti-Fc blot on the intracellular protein fractions (Figure 4b), revealed the presence of smeary signals around 50 kDa and 100 kDa, likely corresponding to proteins lacking or with improper disulphide bonds. The apparent MW of 50 kDa of the intracellular monomer species also suggests that the protein is not or partially glycosylated (as opposed to the monomer of 59 kDa shown in Figure 2). These signals were weaker for WT RBDmfc (Figure 4a, lane 1) than for B.1.351 RBDmfc (Figure 4a, lane 4), in agreement with the better secretion of WT vs. B.1.351 RBDmfc proteins. Upon overexpression of PDIA2, the intensity of these two smeary bands was enhanced for both WT and B.1.351 RBDmfc (Figure 4b, anti-Fc blot, lanes 2 and 3 for WT, lanes 5 and 6 for B.1.351), demonstrating the intracellular accumulation of proteins with improper disulphide bonds (and incomplete glycosylation). Comparable results were obtained using anti-RBD antibodies (Figure 4b), albeit with weaker signals due to the poor detection of intracellular RBD proteins by this monoclonal antibody pool (see Section 2.2.2 Materials and Methods).

Altogether, these results demonstrate that WT and B.1.351 RBDmfc present distinct disulphide bond arrangements, likely resulting in misfolding and enhanced intracellular retention of a portion of B.1.351 RBDmfc proteins. The observation that the protein disulphide isomerase PDIA2 exacerbated the accumulation of proteins with improper disulphide bonds further supports the idea that aberrant disulphide bond arrangements are responsible for the retention of B.1.351 RBDmfc in the ER (in the absence of PDIA2 overexpression).

Aggregation of misfolded proteins in the ER is responsible for the ER-stress response UPR [23,42,49]. One of the outcomes of UPR is the induction of chaperones, meant to promote protein folding. Western blot analysis revealed an increase in the protein level of endogenous PDIA2 (Figure 4b, *), HSPA5, and to a lesser extent HSP90B1, in cells expressing B.1.351 RBDmfc compared to WT RBDmfc (Figure 4b, lanes 4–6 vs. lane 1). HSPA5 (and to a lesser extent HSP90B1) also appeared upregulated in WT RBDmfc cells overexpressing PDIA2 (Figure 4b, lanes 2 and 3 vs. lane 1). These results suggest that the UPR was induced in cells expressing B.1.351 RBDmfc, likely as a response to the ER accumulation of misfolded mutant proteins.

### 3.4. Glycosylation Profile of Intracellular and Secreted WT and B.1.351 RBDmfc Proteins

**Figure 5:**
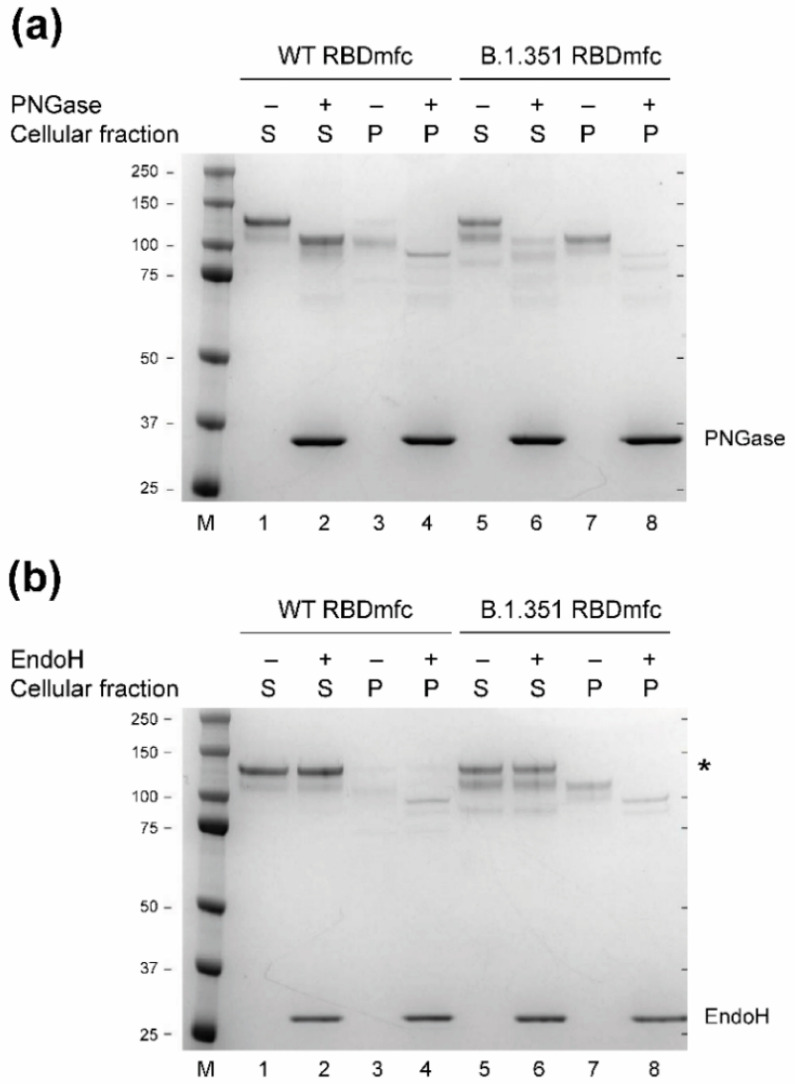
SDS-PAGE and Coomassie staining of purified RBDmfc proteins (S, secreted in culture medium; P, lysate from cell pellet, representing the intracellular fraction) before and after deglycosylation by PNGase (**a**) or EndoH (**b**). M, protein ladder (apparent MW in kDa indicated on the left); *, upper MW form (apparent MW of 120 kDa). Uncropped SDS-PAGE images are shown in Appendix A Figure A2.

To determine whether intracellular B.1.351 RBDmfc proteins indeed accumulate into the ER and do not transit to the Golgi apparatus, we investigated the sensitivity of intracellular and secreted RBDmfc proteins to the deglycosylases PNGase and EndoH. PNGase deglycosylates all N-glycosylations, thus glycosylated proteins are expected to be sensitive to PNGase as they proceed through both the ER and Golgi apparatus. On the other hand, glycosylated proteins are expected to lose their sensitivity to EndoH during maturation in the Golgi apparatus. Purified secreted and intracellular WT and B.1.351 RBDmfc proteins were treated with PNGase or EndoH and analysed by SDS-PAGE and Coomassie staining (Figure 5). RBDmfc proteins, whether WT or mutant, secreted or intracellular, were sensitive to PNGase, resulting in a downward shift (Figure 5a, lanes 2, 4, 6, 8 vs. 1, 3, 5, 7, respectively), indicating that all proteins were glycosylated. As expected, secreted proteins, both WT and B.1.351 RBDmfc, were resistant to EndoH (Figure 5b, lane 1 vs. 2 for WT and lane 5 vs. 6 for B.1.351). The intracellular forms of WT and B.1.351 RBDmfc were sensitive to EndoH (Figure 5b, lane 3 vs. 4 for WT and lane 7 vs. 8 for B.1.351), demonstrating that they reside in the ER. Interestingly, a faint upper MW band (~120 kDa), corresponding to the main secreted form, was detected in WT RBDmfc-expressing cells and was resistant to EndoH (Figure 5b, lanes 3 and 4, *). This band likely represents intracellular WT RBDmfc proteins in transit through the Golgi along the secretory pathway. Such a band was not detected in B.1.351 RBDmfc-expressing cells (Figure 5b, lanes 7 and 8, *), probably due to its lower abundance compared to WT RBDmfc (see Figure 2). These results confirmed that the pool of RBDmfc proteins detected intracellularly is retained in the ER, and is likely prevented from proceeding to secretion because of improper disulphide bonds and misfolding.

Altogether, these data demonstrate that, compared to WT RBDmfc, only a small fraction of B.1.351 RBDmfc proteins, presenting the correct disulphide bond arrangements and proper folding, is secreted from transfected Expi293F cells, while a pool of misfolded proteins is retained in the ER, resulting in the UPR ER-stress response.

### 3.5. Hydrophobicity and Thermostability of WT and B.1.351 RBDmfc Fusion Proteins

**Figure 6:**
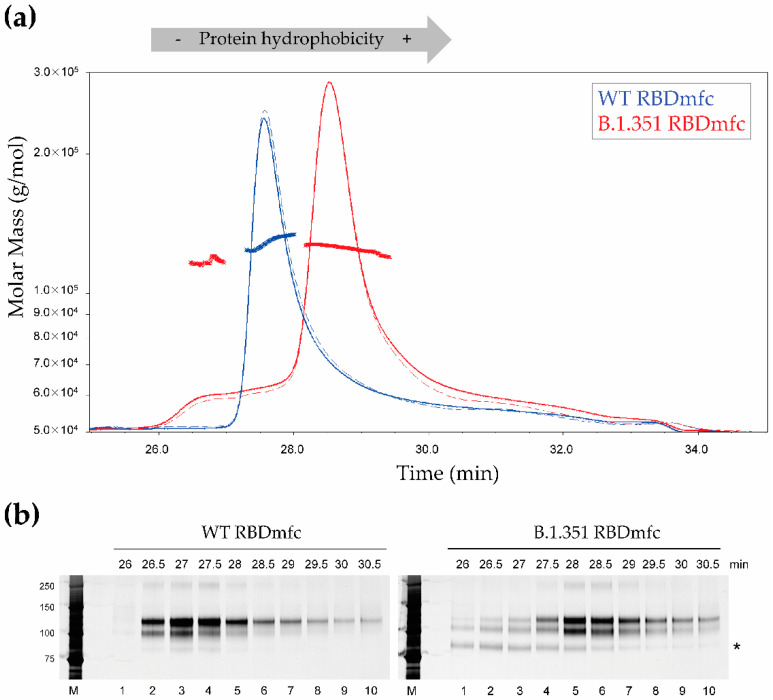
Hydrophobicity plot analysis of WT and B.1.351 RBDmfc proteins. (**a**) Hydrophobicity plots were determined by hydrophobic interaction chromatography hyphenated to multi-angle light scattering detection (HIC-MALS). Plain curves represent UV_280 nm_ absorbance (for protein concentration determination) and dotted curves represent scattered light intensity (determined by MALS). WT and B.1.351 RBDmfc protein populations show different hydrophobicity profiles, as expected from their differences in amino acid composition. However, B.1.351 but not WT RBDmfc presents a second protein population with lower hydrophobicity (see shoulder for fractions collected between 26 and 28 min). Absolute molar mass calculations (based on the light scattering theory) are depicted as a bold line across each peak, as well as above the lower hydrophobicity B.1.351 protein population eluting between 26 and 28 min. Calculated molar mass indicates a relative homogeneity in molar mass between the different protein populations. (**b**) SDS-PAGE and silver staining of purified, secreted RBDmfc proteins fractionated by HIC-MALS [see panel (**a**)]. M, protein ladder (apparent MW in kDa indicated on the left); *, protein population with lower apparent MW. Fractions were named as follows: Fraction “26 min” corresponds to the fraction collected between 26 and 26.5 min in panel (**a**), fraction “26.5 min” to the fraction collected between 26.5 and 27 min, etc. Uncropped SDS-PAGE images are shown in Appendix A Figure A5.

To further characterise WT and B.1.351 RBDmfc proteins, purified secreted proteins were fractionated according to their hydrophobicity status using hydrophobic interaction chromatography hyphenated to multi-angle light scattering detection (HIC-MALS). WT and B.1.351 RBDmfc protein preparations exhibited different hydrophobicity profiles (Figure 6a), as expected from their differences in amino acid composition. As opposed to WT RBDmfc, which eluted as one main peak (at 27–29 min), B.1.351 RBDmfc revealed two protein populations (eluting at 26–28 min and 28–30 min, respectively), thus presenting differences in hydrophobicity (Figure 6a).

**Figure 7:**
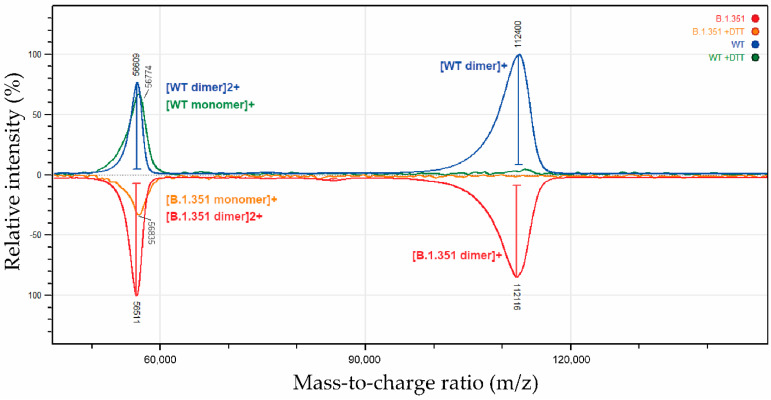
Intact mass analysis of WT and B.1.351 RBDmfc proteins by MALDI-TOF. Intact mass of non-deglycosylated RBDmfc proteins was determined on secreted WT and B.1.351 RBDmfc recombinant proteins, as well as on their DTT-reduced counterparts. The mass-to-charge ratio (*m*/*z*) was plotted against the relative abundance of fragment ions (relative intensity [%]) for WT (top panel, blue and green) and B.1.351 (bottom panel, red and orange) RBDmfc proteins. Doubly charged ions (marked “2+“, compared to singly charged ions marked “+“) appear at “half mass“. Mass spectrometry confirmed the homogeneity in molar mass characterised by HIC-MALS (Figure 6) and by SEC (Figure 8) for WT and B.1.351 RBDmfc proteins.

**Figure 8:**
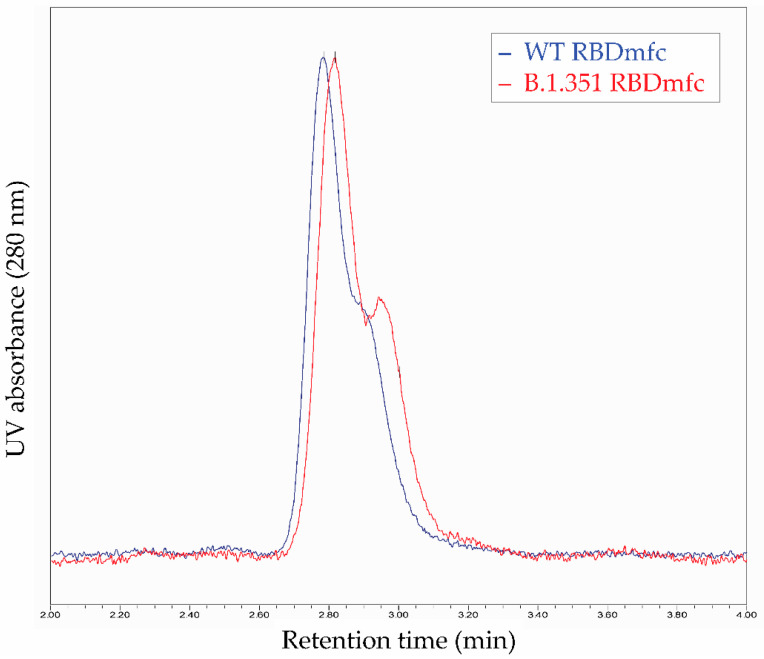
Size-exclusion chromatography (SEC) of WT and B.1.351 RBDmfc proteins. SEC was conducted on secreted, purified WT and B.1.351 RBDmfc recombinant proteins. UV absorbance was monitored at 280 nm and plotted against the retention time. Retention time of both proteins was similar: 2.79 and 2.82 min for WT and B.1.351 RBDmfc proteins, respectively. SEC thus confirmed the homogeneity in molar mass of WT and B.1.351 RBDmfc recombinant proteins, in agreement with HIC-MALS (Figure 6) and intact mass spectrometry (Figure 7) data.

MALS analysis confirmed similar absolute molar mass for the different protein populations (Figure 6a). This homogeneity in molar mass was confirmed by intact mass spectrometry analysis using MALDI-TOF (Figure 7) and by size-exclusion chromatography (Figure 8). Moreover, the 56 kDa mass observed for monomers under reducing conditions by intact mass spectrometry (Figure 7, “+DTT“) also confirmed the presence of post-translational modifications (such as glycosylation) that occurred during expression in HEK293 cells, resulting in increased molecular weight compared to the predicted 49 kDa of RBDmfc monomers (see Figure 1).

SDS-PAGE and silver staining analysis of the fractions isolated by HIC-MALS revealed that the two main peaks in WT and B.1.351 RBDmfc preparations corresponded to the protein population with higher apparent MW (~105 and 120 kDa) (Figure 6b, lanes 2–5 of left panel and lanes 4–7 of right panel). On the other hand, the B.1.351 RBDmfc protein population with lower hydrophobicity corresponded to the population with lower apparent MW (~85 kDa) (Figure 6b, see * in lanes 1–4 of right panel). The observation that a fraction of B.1.351 RBDmfc proteins with similar molar mass exhibited a lower apparent MW on SDS-PAGE, together with a lower hydrophobicity, strongly suggests that this protein population presents a different conformation compared to the proteins with higher apparent MW.

**Figure 9:**
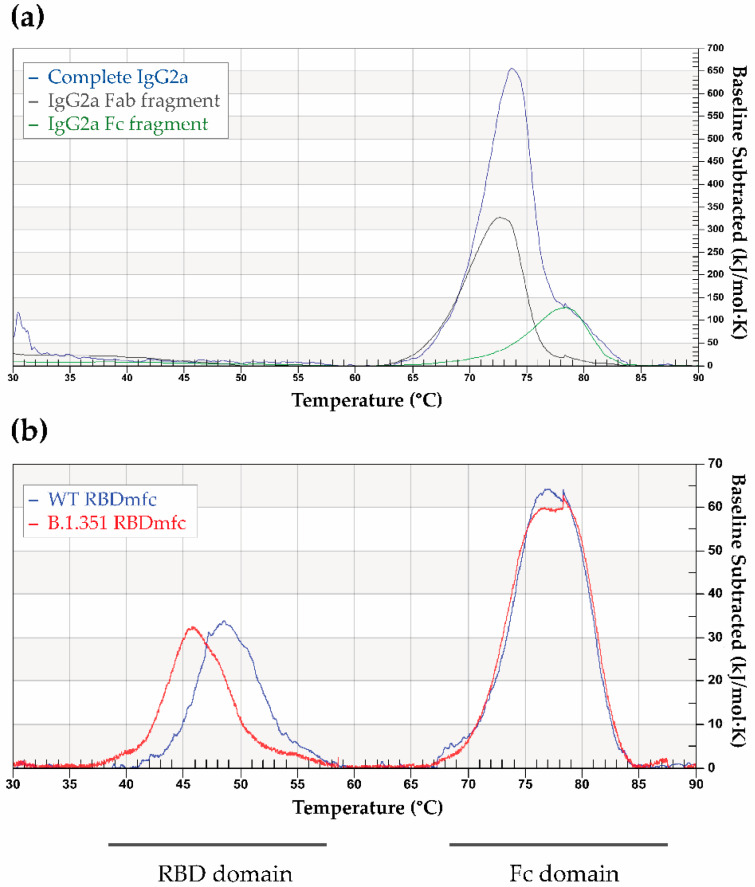
Thermograms of mouse IgG2a Fc fragment (**a**) and of WT and B.1.351 RBDmfc proteins (**b**) acquired by differential scanning calorimetry (DSC). (**a**) DSC was performed on purified mouse IgG2a and on its Fab and Fc fragments obtained upon papain digestion. Mouse IgG2a Fc fragment shows a Tm around 77–78 °C. (**b**) DSC was performed on purified WT and B.1.351 RBDmfc proteins. The Tm of the Fc domain of both proteins overlaps around 77 °C. The RBD domains of WT and B.1.351 recombinant proteins differ in their respective Tm, indicating a reduced thermostability of the RBD domain of B.1.351 RBDmfc vs. that of WT RBDmfc.

Finally, thermograms of the purified WT and B.1.351 RBDmfc secreted proteins were established by differential scanning calorimetry (DSC). After baseline subtraction and integration of the signals, each tested variant showed two independent denaturation events at a half-denaturation temperature (Tm) of approximatively 46–49 °C and 77 °C, respectively (Figure 9b). Independent DSC experiments using mouse IgG2a and its Fab and Fc fragments showed that the thermal denaturation event occurring at Tm 77 °C corresponded to the Fc domain (Figure 9a, green curve), and consequently that the first denaturation event at Tm 46–49 °C was related to the RBD subdomain (Figure 9b).

Tm and reaction enthalpy (ΔH) were measured from the thermograms for the two denaturation events and compared between WT and B.1.351 RBDmfc. Tm1 and ΔH1 measurements for the two RBD substructures highlighted a higher Tm and ΔH for WT RBD (48.6 °C and 56.0 kcal/mol, respectively) than for B.1.351 RBD (45.9 °C and 52.0 kcal/mol, respectively), whereas Tm2 and ΔH2 for the Fc domain of both RBDmfc proteins showed similar thermodynamical values (Tm2[WT] = 77.2 °C; Tm2[B.1.351] = 77.3 °C; ΔH2[WT] = 121.7 kcal/mol; ΔH2[B.1.351] = 122.0 kcal/mol).

Altogether, the DSC thermograms indicate that the RBD domain of the B.1.351 mutant presented a reduced thermostability compared to the WT RBD domain, further supporting the idea that WT and B.1.351 RBDmfc proteins exhibit different conformations, and that B.1.351 altered conformation involves the RBD subdomain of the RBDmfc fusion protein.

## 4. Discussion

We investigated the impact of naturally occurring SARS-CoV-2 RBD mutations on the production and secretion of a recombinant Fc-tagged RBD protein (RBDmfc) transiently expressed in HEK 293 cells. We showed that mutation E484K and the association E484K+K417N, but not N501Y, greatly impaired the secretion of RBDmfc. The comparison of WT and B.1.351 RBDmfc proteins (the latter carrying mutations K417N, E484K and N501Y) demonstrated that impaired secretion of the B.1.351 RBDmfc was not due to a defect in protein translation but to its retention in the ER. WT and B.1.351 RBDmfc proteins showed differences in disulphide bond arrangements, hydrophobicity and thermostability, suggesting that both proteins differ in their conformation and folding. We, therefore, propose that improper disulphide bond formation and misfolding are responsible for the diminished secretion of B.1.351 RBDmfc, compared to WT RBDmfc, upon expression in HEK 293 cells.

We attempted to improve B.1.351 RBDmfc protein folding and secretion by co-expressing the chaperone and protein disulphide isomerases PDIA2, as successfully described for other mutated proteins [46,47]. Unexpectedly, PDIA2 overexpression resulted in the increased retention of both WT and B.1.351 RBDmfc proteins with altered disulphide bonds in the ER. Such “anti-chaperone” behaviour of PDIA2 has been previously reported [42,58,59]. The mechanisms controlling the chaperone vs. anti-chaperone activities of PDIA2 are not fully understood, but some evidence suggests that PDIA2′s anti-chaperone activity might preferentially target disulphide-rich proteins under certain cellular conditions [58,59]. Interestingly, our results resemble those reported by Davis et al. [58], demonstrating that overexpression of PDIA2 in mammalian cells was associated with the ER retention of the recombinant tumour necrosis factor receptor (TNFR)-Fc fusion protein (containing 14 disulphide bridges), but not of the globular protein IL-15 (containing two disulphide bonds) [58]. These authors showed that retention of TNFR-Fc in the ER by PDIA2 required the disulphide isomerase activity of PDIA2 [58]. These observations suggest that upon overexpression of PDIA2 in HEK 293 cells, RBDmfc proteins are retained in the ER and their disulphide bonds are rearranged by PDIA2. In the process, WT and B.1.351 RBDmfc proteins’ disulphide bonds are differentially altered, likely due to differences in WT and B.1.351 protein folding.

In addition, the observation that the intracellular level of B.1.351 RBDmfc was further increased upon overexpression of PDIA2 suggests that aggregated misfolded B.1.351 RBDmfc might be normally eliminated, possibly via the ER-associated protein degradation (ERAD) pathway, in the absence of exogenous PDIA2, in order to preserve ER integrity and homeostasis [23,44,49]. This further support the idea that the overall lower yield of B.1.351 RBDmfc vs. WT RBDmfc was not due to a defect in protein translation but rather in trafficking along the secretory pathway.

The different disulphide bond arrangements detected between WT and B.1.351 RBDmfc are likely due to differences in protein conformation and folding caused by point mutations within B.1.351 RBD, especially E484K, and to a lesser extent K417N. This proposition is in agreement with the observations that mutation E484K affects the structure and conformation of the spike protein [54,55,56] and that disulphide bond formation plays an essential role in the structure and function of the RBD [53]. These differences in protein folding might be responsible for the improper disulphide bond formation, which is known to be influenced by the proximity and accessibility of cysteine residues [38,41]. These differences in protein structure were also evidenced by the hydrophobicity profile of the WT and B.1.351 proteins and might explain the reduced thermostability of the RBD domain of the B.1.351 RBDmfc fusion protein identified by differential scanning calorimetry, in line with the important role of disulphide bond formation in protein structure stabilisation [42,53]. The reduced thermal stability of the B.1.351 RBD domain compared to that of WT RBD detected in our study is in agreement with a previous report demonstrating the destabilising effect of E484K and K417N mutations, but not of the N501Y mutation, in thermal denaturation experiments [60].

Finally, the critical role of residue E484K was recently confirmed in our laboratory by preliminary experiments performed using an RBDmfc variant based on the recently emerged SARS-CoV-2 omicron (B.1.1.529 or BA.1) variant [19]. Among the 15 RBD mutations of the B.1.1.529 variant, three residues 417, 484 and 501 are also mutated in the B.1.351 variant. Although mutations at residues 417 and 501 are shared by both variants (K417N, N501Y), the amino acid substitution at the critical residue 484 differs (E484A in B.1.1.529 vs. E484K in B.1.351). Interestingly, the secretion yield of B.1.1.529 RBDmfc in 293F cells was improved compared to that of B.1.351 RBDmfc, being almost comparable to that of WT RBDmfc (Figure S3). The E484K mutation results in the substitution of a negatively charged amino acid (glutamic acid E) with a positively charged amino acid (lysine K), while the E484A mutation reverses the positively charged K to a neutral and hydrophobic amino acid (alanine A). This modification is likely to alter electrostatic interactions and the conformation of the RBD [55], as further suggested by the 3D structure comparison surrounding amino acid 484 (Figure S4). Although we cannot exclude an additional impact of the 12 other RBD mutations in the production yield of B.1.1.529 RBDmfc, these preliminary data confirm the importance of amino acid 484 for the proper folding and secretion of RBD recombinant proteins.

It is worth noting that in the context of its C-terminal fusion with the Fc domain, our recombinant RBD protein allows principally only three of the four disulphide bonds identified in the native RBD (namely C336-C361, C379-C432 and C480-C488) [51,52,53]. Indeed, in our fusion protein, the RBD domain ends at amino acid 524, thus preventing the formation of the native disulphide bond C391-C525. Other recombinant Fc-tagged RBD proteins (amino acids 331-524), thus lacking C525, were previously described as retaining structural and biological properties of the native RBD domain, such as efficient binding to the human ACE2 receptor [14,15,16]. Here, we additionally demonstrated the antigenic property of RBDmfc, by showing its ability to be recognised by SARS-CoV-2-specific IgG from COVID-19 convalescent patients, in agreement with previous results based on the His-tagged version of recombinant RBD (amino acids 331-524) [13]. The missing C391-C525 disulphide bond did not impede the efficient production and secretion of WT RBDmfc. However, in the context of the B.1.351 mutant and its altered conformation, we cannot exclude that the lack of this amino acid might contribute to the reduced efficiency of protein secretion, by further affecting disulphide bond formation and isomerization during protein maturation in the ER. Additional experiments would be needed to address this question. Finally, the data presented here should serve as a basis for the design of optimisation protocols aiming to improve the production of B.1.351 RBDmfc and other difficult-to-express variant proteins, for instance by manipulating the UPR pathway, as described for unrelated recombinant proteins [23,24,33,34].

## 5. Conclusions

We showed that RBD mutation E484K has a major impact on the folding, disulphide bond formation and secretion of a recombinant RBD protein expressed in human 293F cells. Optimisation protocols, possibly manipulating the UPR, might permit us to overcome this challenge.

## Figures and Tables

**Figure A1:**
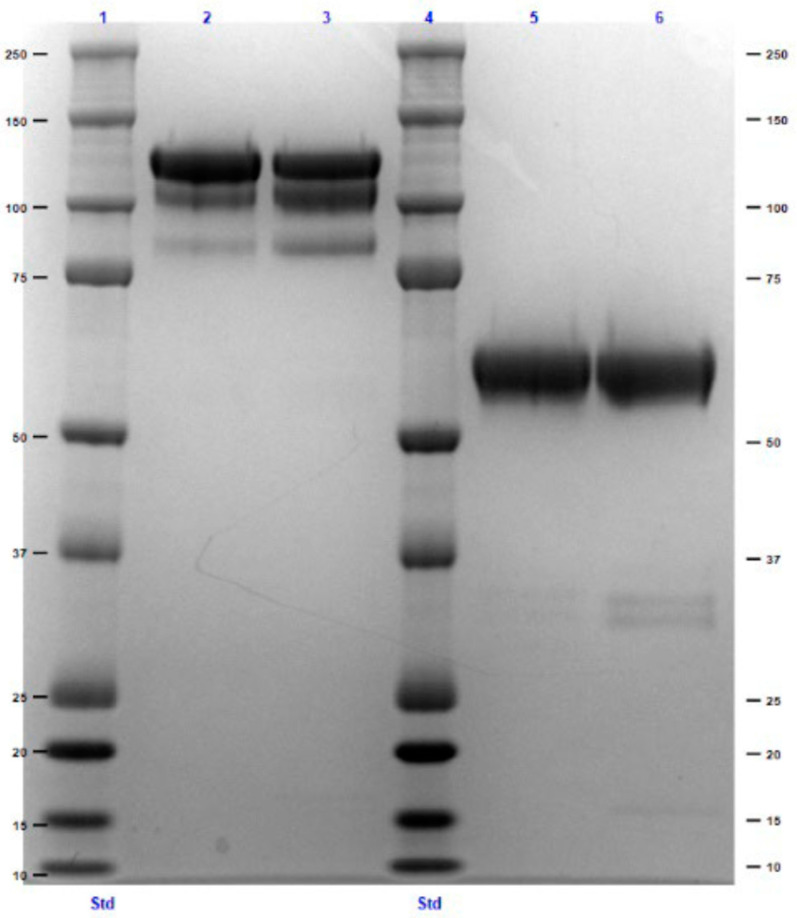
UncroppedCoomassie-stainedgel shownin Figure 2.

**Figure A2:**
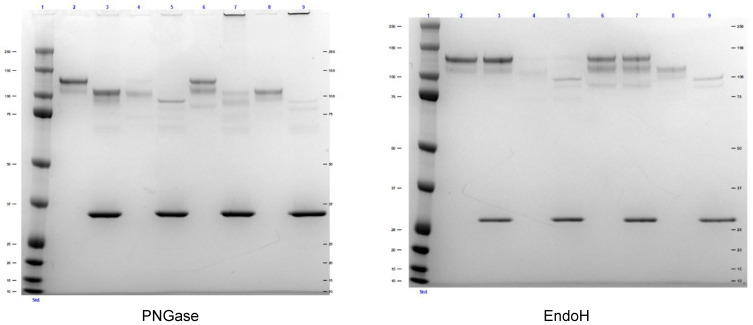
UncroppedCoomassie-stainedgel shownin Figure 5.

**Figure A3:**
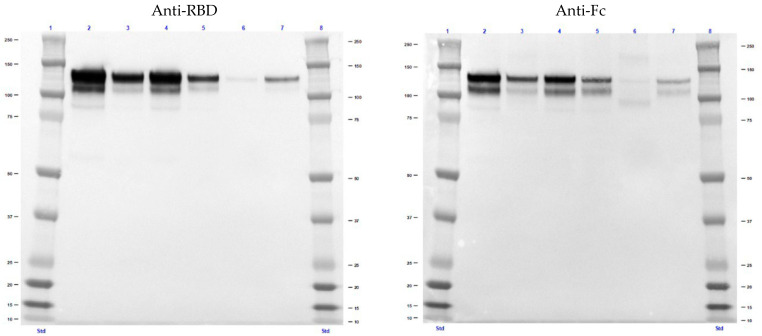
Uncropped western blot images shown in Figure 4a (secreted proteins).

**Figure A4:**
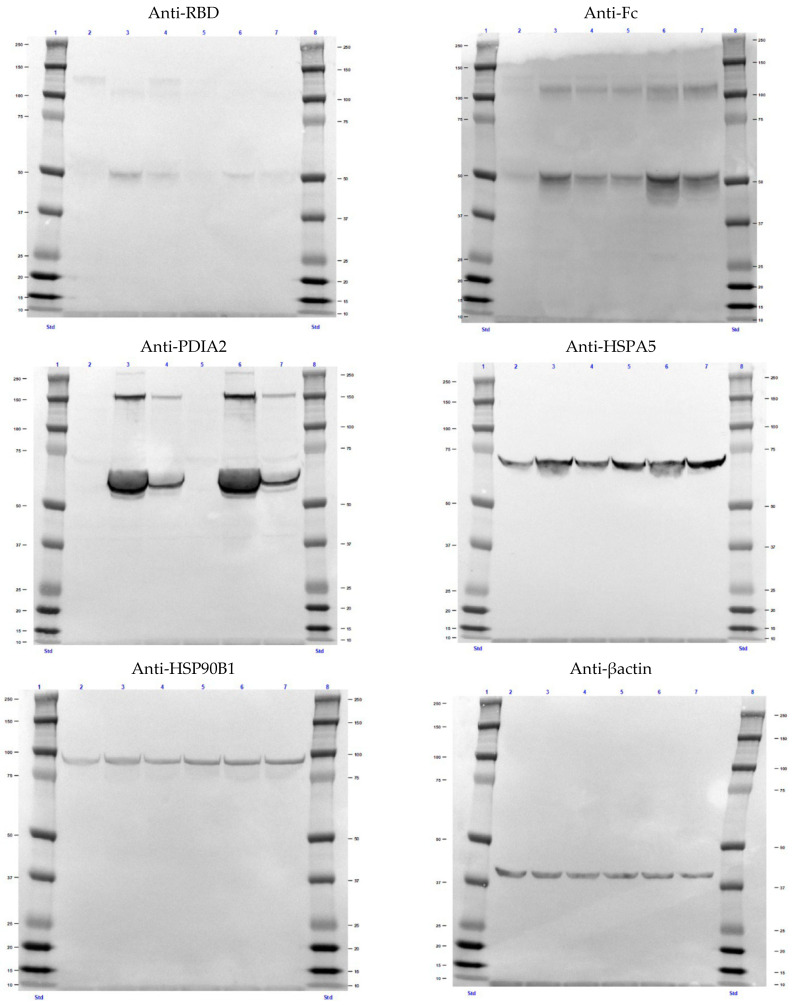
Uncropped western blot images shown in Figure 4b (intracellular proteins).

**Figure A5:**
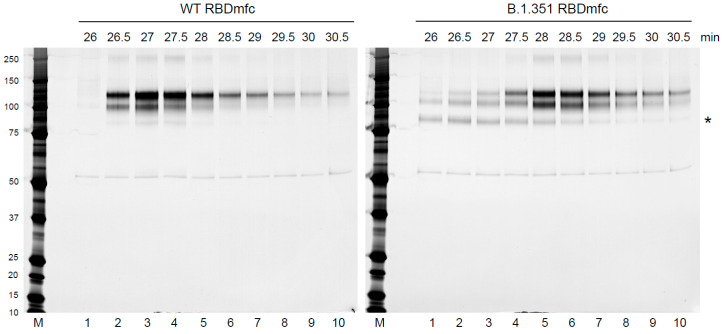
Uncroppedsilver-stainedgel shownin Figure 6.

## Data Availability

The data presented in this study are available within the article and supplementary materials.

## Supplementary Materials

The following supporting information can be downloaded at: https://www.mdpi.com/article/10.3390/biom12091170/s1, Figure S1: Functionality of purified secreted WT and B.1.351 RBDmfc recombinant proteins; Figure S2: Quantification of Western blot signal intensity; Figure S3: Expression of recombinant WT and B.1.1.529 RBDmfc proteins in Expi293F cells.

## Appendix A

Uncropped SDS-PAGE images.
