# Supplementary material for: Impact of SARS-CoV-2 RBD Mutations on the Production of a Recombinant RBD Fusion Protein in Mammalian Cells
Source: Biomolecules. 2022 Aug 24;12(9):1170. doi: 10.3390/biom12091170 (PMC9496381; doi:10.3390/biom12091170)
Supplement: Supplementary file 1 [file biomolecules-12-01170-s001.zip › biomolecules-1866016-supplementary.pdf]

(a)

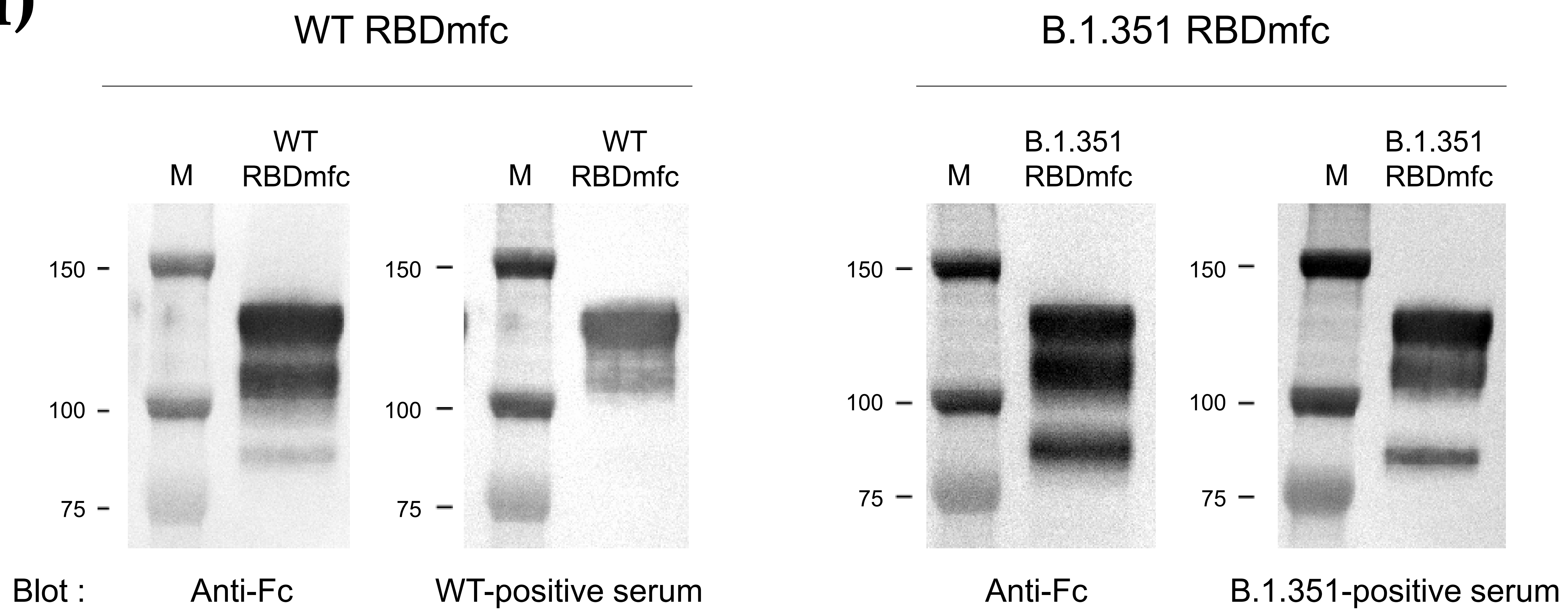

Relative band intensity (%)	
Band 1	54.1
Band 2	36.5
Band 3	9.4

Relative band intensity (%)	
Band 1	87.9
Band 2	12.1
Band 3	-

Relative band intensity (%)	
Band 1	39.2
Band 2	38.5
Band 3	22.3

Relative band intensity (%)	
Band 1	54.5
Band 2	28.2
Band 3	17.3

(b)

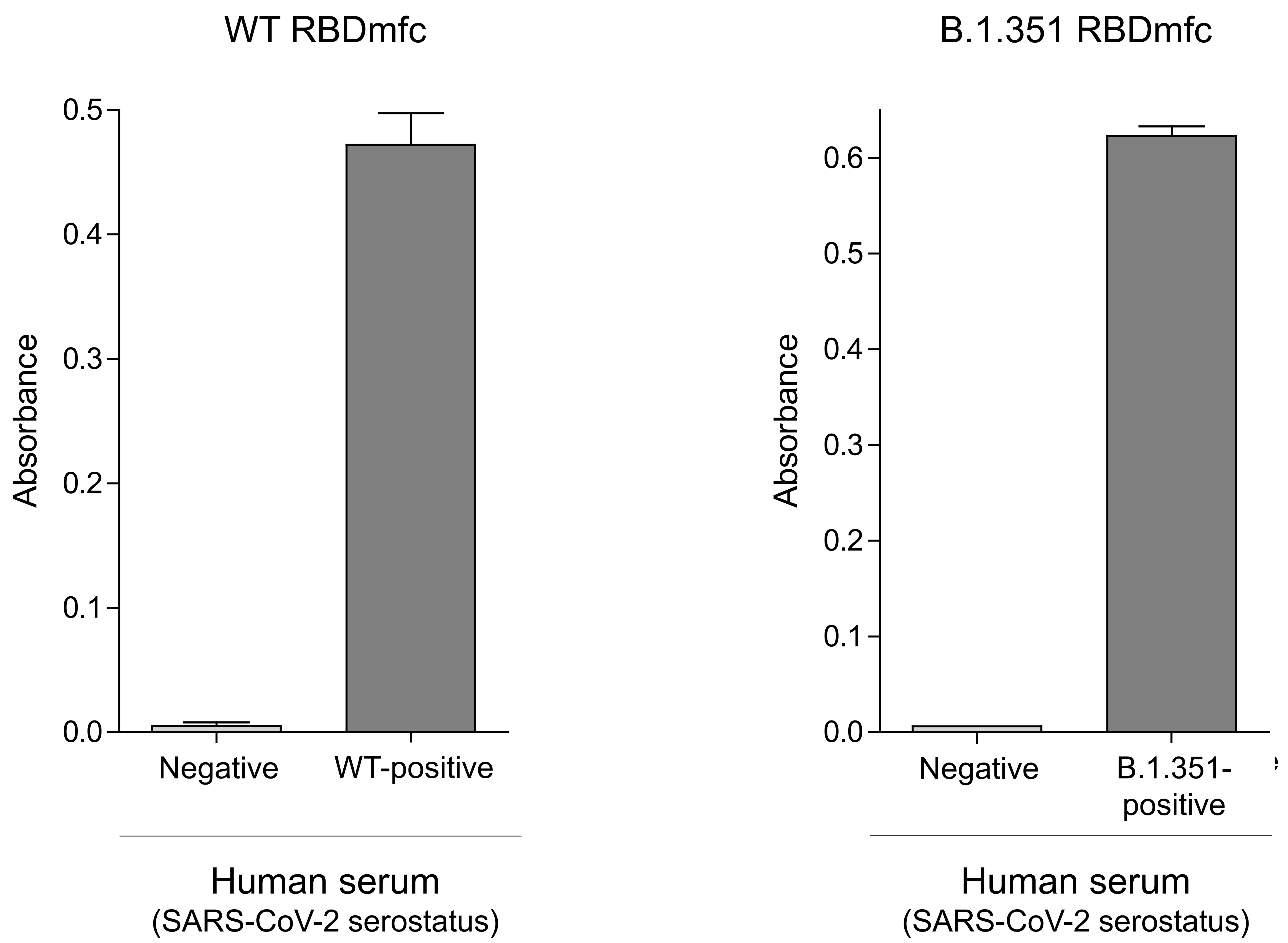

Figure S1. Functionality of purified secreted WT and B.1.351 RBDmfc recombinant proteins. The ability of recombinant RBDmfc to be recognized by RBD-specific antibodies was evaluated by Western-blot (a) and enzyme-linked immunosorbent assay (ELISA) (b). **(a)** WT and B.1.351 proteins were separated by SDS-PAGE. Western-blotting with an anti-Fc antibody (used as positive control) detected three main bands with an apparent molecular weight of 120, 105 and 85 kDa. The upper band signal was predominant, especially for WT RBDmfc (54.1%) *vs.* B.1.351 RBDmfc (39.2%). Human sera of patients infected with WT or B.1.351 SARS-CoV-2 recognized the respective recombinant RBDmfc proteins. The higher molecular weight form of both RBDmfc proteins was more strongly detected by the respective sera (87.9% for WT and 54.5% for B.1.351). M, protein ladder (kDa). **(b)** ELISA was conducted on coated WT or B.1.351 RBDmfc proteins, using either a human serum from a pre-pandemic donor (negative control) or human sera of patients infected with WT or B.1.351 SARS-CoV-2. In both cases, signal intensity for SARS-CoV-2-specific sera was about 100-fold over that of the negative control.

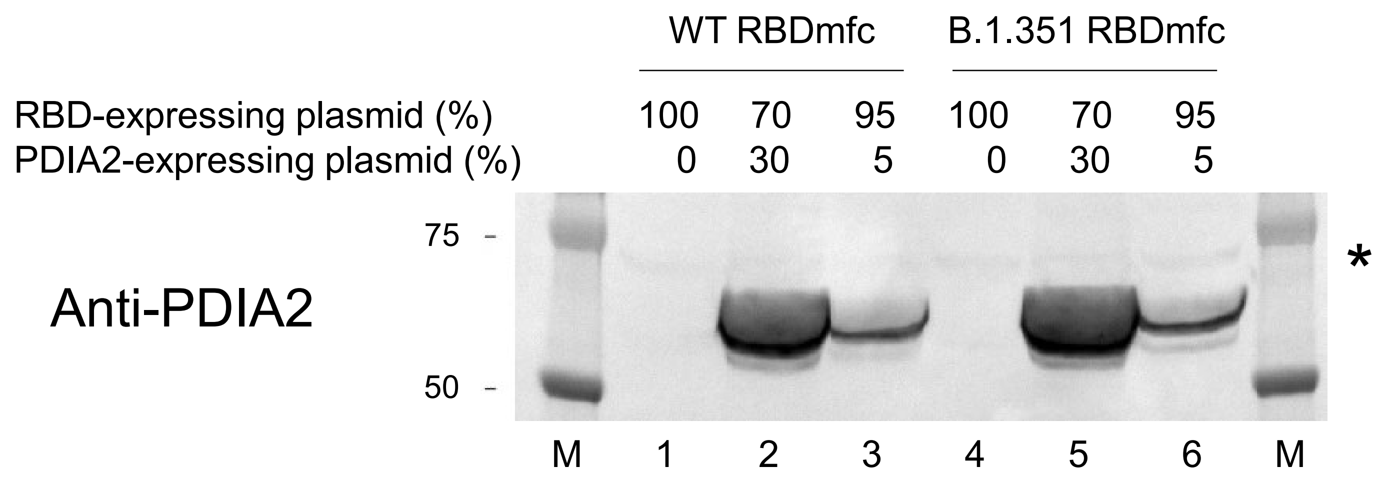

Relative band intensity								
Endogenous PDIA2 (*)	-	**1.00**	0.84	0.43	2.85	3.14	2.58	-
Exogenous PDIA2	-	-	224.3	77.3	-	262.1	83.2	-

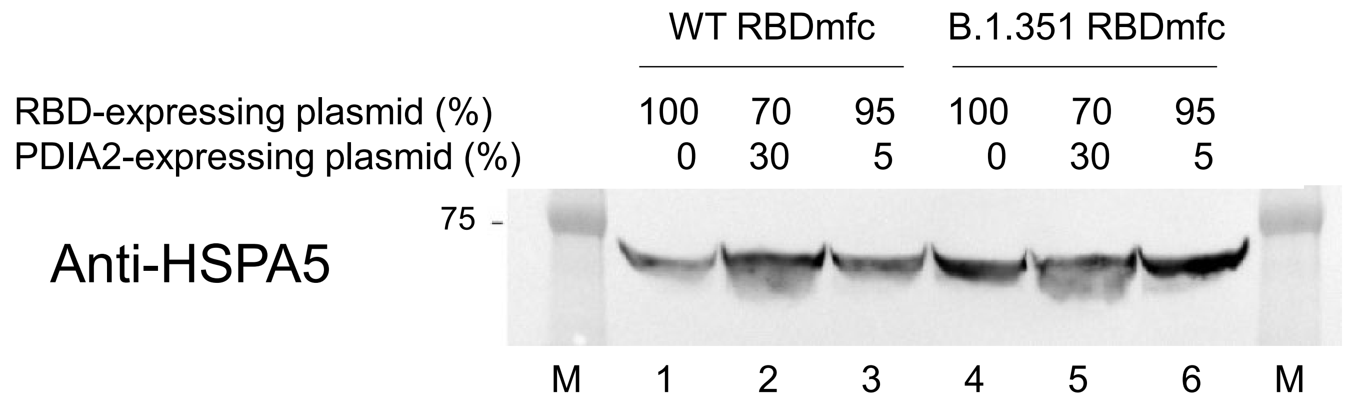

Relative band intensity	-	**1.00**	2.84	1.48	2.10	2.52	2.72	-

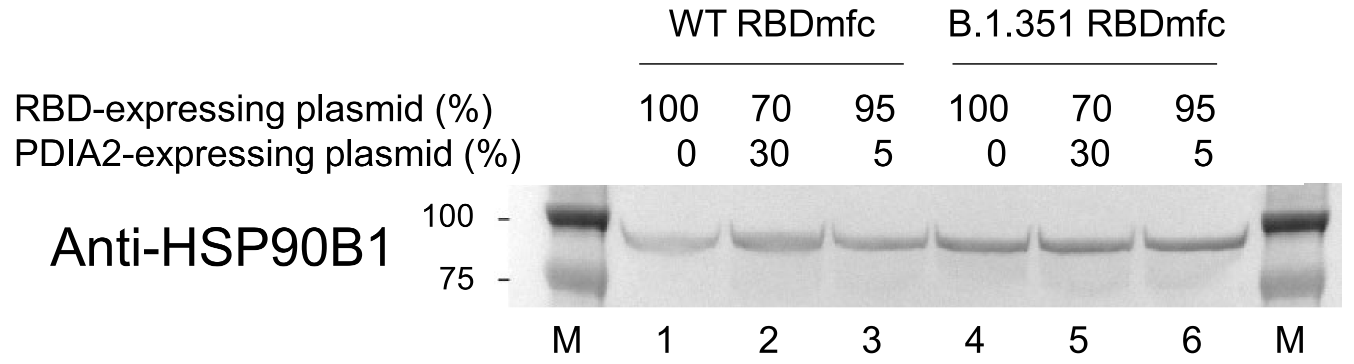

Relative band intensity	-	**1.00**	1.34	1.00	1.48	1.56	1.70	-

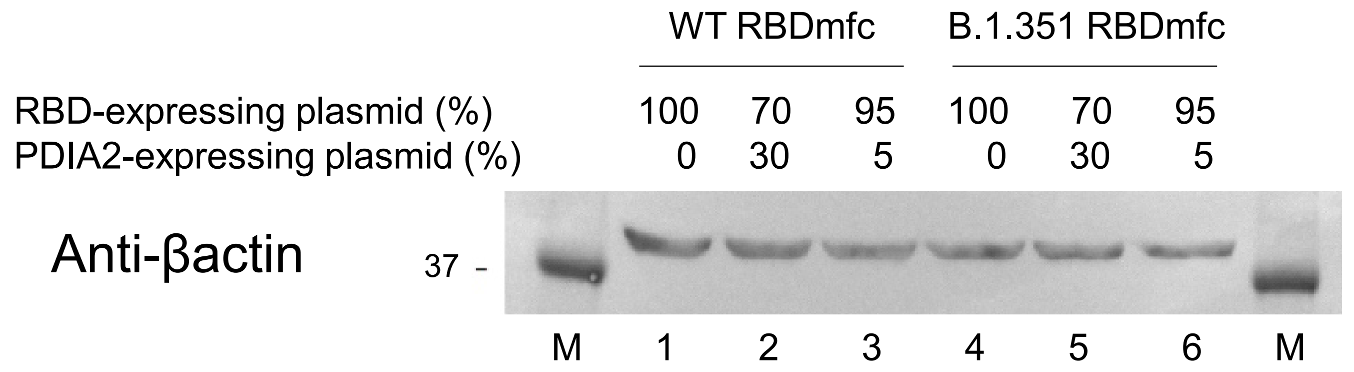

Relative band intensity	-	**1.00**	0.80	0.70	0.97	0.81	0.78	-

Figure S2. Quantification of Western blot signal intensity. Quantification of signal intensity detected for chaperones (PDIA2, HSPA5, HSP90B1) and βactin (as negative control) relative to the condition in 100% WT RBDmfc (lane 1 = 1.00) (see Figure 4b). M, protein ladder (kDa).

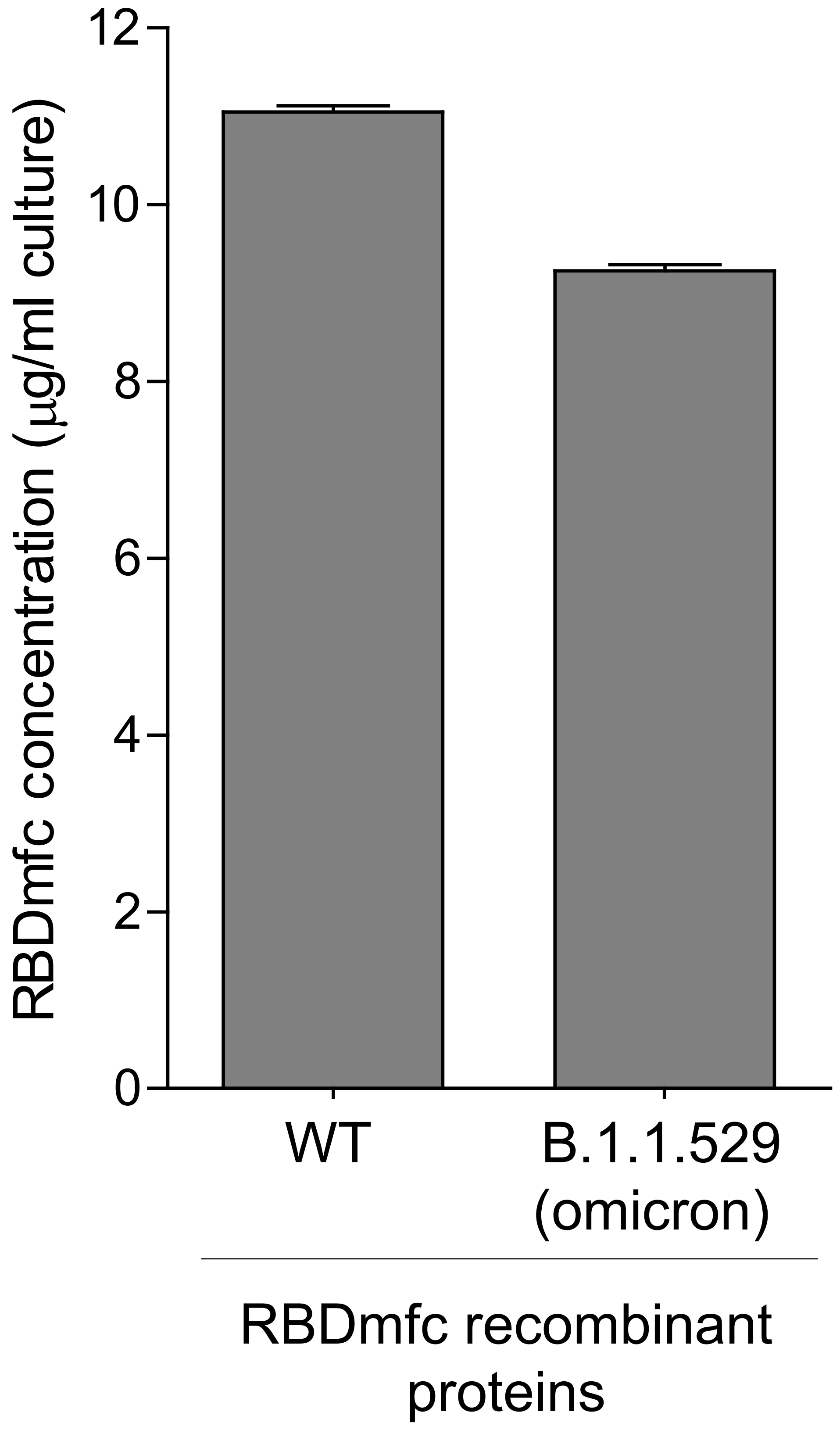

Figure S3. Expression of recombinant WT and B.1.1.529 RBDmfc proteins in Expi293F cells. Concentration of secreted RBDmfc proteins in the supernatant of Expi293F cells, measured by Octet RED96 and expressed in µg/ml culture. Values are mean (standard deviation) of duplicate Octet measurements from one transfection experiment. The RBDmfc recombinant protein carrying omicron B.1.1.529 (BA.1) mutations (15 mutations compared to WT RBD) was expressed at a slightly lower level than WT RBDmfc (83% of WT) in the supernatant of Expi293F cells. This contrasts with the strongly reduced level of secreted B.1.351 RBDmfc recombinant protein (27% of WT; Figure 1b). B.1.1.529 RBDmfc carries mutations at the three residues 417, 484 and 501, in addition to 12 other mutations. Mutations K417N and N501Y are shared with B.1.351 RBDmfc while mutation at amino acid 484 differs between both variants (E484K in B.1.351 RBDmfc *vs.* E484A in B.1.1.529).

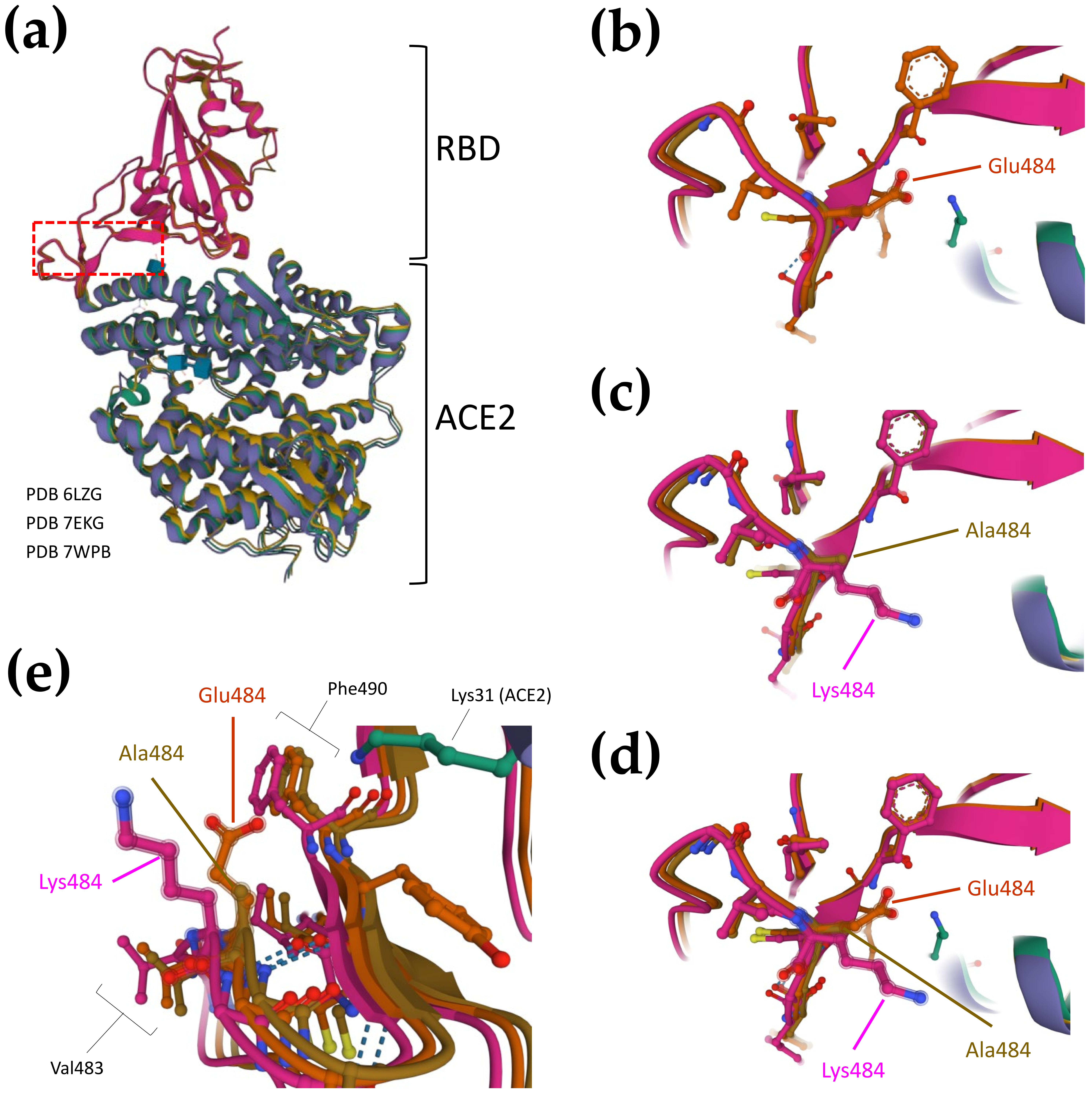

Figure S4. 3D structures of SARS-CoV-2 RBD WT, B.1.351 and B.1.1.529. 3D structural superposition of WT, B.1.351 (beta) and B.1.1.529 (omicron) RBD in complex with ACE2, obtained from RCSB PDB (https://www.rcsb.org) and generated using Mol* Viewer (Sehnal et al. 2021, Nucleic Acids Res. 49(W1):W431-W437, doi: 10.1093/nar/gkab314). The respective PDB identification code and structure publication are as follows. RBD WT: PDB 6LZG, Wang et al. 2020, Cell 181:894-904.e9; RBD B.1.351: PDB 7EKG, Han et al. 2021, Nat Commun 12:6103-6103; RBD B.1.1.529: PDB 7WPB, Yin et al. 2022, Science 375:1048-1053.
(**a**) Superimposed 3D structures of RBD-ACE2 complexes, showing overall comparable structures of WT, B.1.351 and B.1.1.529 RBD in complex with ACE2. The region surrounding amino acid 484 of RBD, which is zoomed in panels (b-d) is framed by a red dotted line. (**b**) Glutamic acid (Glu) 484 (E484) in WT RBD. (**c**) Lysine (Lys) 484 (mutation E484K of variant B.1.351) and alanine (Ala) 484 (mutation E484A of variant B.1.1.529). (**d**) Superposition of WT E484 with E484K and E484A mutations of the B.1.351 and B.1.1.529 variants, showing the different position of E484K compared to E484A and to the WT residue E484. (**e**) Different perspective of the 3D superposition shown in panel (d), highlighting the different position of the positively charged lysine (E484K) of B.1.351 RBD, compared to the negatively charged glutamic acid (E484) of WT RBD and the neutral and hydrophobic alanine (E484A) of B.1.1.529 RBD. By contrast, surrounding amino acids are well superimposed.
